# The UK clinical eye research strategy: refreshing research priorities for clinical eye research in the UK

**DOI:** 10.1038/s41433-024-03049-6

**Published:** 2024-05-29

**Authors:** Rupert R. A. Bourne, Malik Moledina, Augusto Azuara-Blanco, George M. Saleh, James E. Self, Sobha Sivaprasad, Srilakshmi M. Sharma, Andrew Ross, Rose M. Gilbert, Maram E. A. Abdalla Elsayed, Won Young Moon, Manjo Doug, Pádraig J. Mulholland, Alexander C. Day, Vito Romano, Geraldine V. Hoad, Madina Kara, Ailish Murray, Louise Gow, Faruque Ghanchi, Praveen J. Patel, Richard P. Gale, Christiana Dinah, Keith Valentine, Cathy Yelf, Vanessa Poustie, NIHR Ophthalmology Specialty Group, Ejaz Ansari, Nick A. V. Beare, Rupert R. A. Bourne, Emma Chambers, Francesca Cordeiro, Samantha De Silva, Susan Downes, Richard Gale, Faruque Ganchi, Chris Hammond, Geraldine V. Hoad, Jonathan Jackson, Anthony King, Andrew J. Lotery, Padraig Mulholland, Ian Nickson, Praveen Patel, Fiona J. Rowe, George M. Saleh, Peter Scanlon, Brinda Shah, Julie Silvestri, Velota Sung, Andrew Tatham, Marta Ugarte, Deepali Varma, Marcela Vortruba, Saila Waseem, Executive Group of UK Clinical Eye Research Strategy, Augusto Azuara Blanco, Michael Bowen, Catey Bunce, Nick Caplin, Roxanne Crosby, Ali Ghareeb, Renata Gomes, Kerry Hanna, Geraldine V. Hoad, Tina Houlihan, Liying Low, James E. Self, Srilakshmi M. Sharma

**Affiliations:** 1 Vision and Eye Research Institute, School of Medicine, Anglia Ruskin University, Cambridge, UK; 2 Department of Ophthalmology, Cambridge University Hospitals, Cambridge, UK; 3 Imperial College NHS Foundation Trust, London, UK; 4 Centre for Public Health, Queen’s University Belfast, Belfast, UK; 5 Research, Cataract and Adnexal Dept, Moorfields Eye Hospital, London, UK; 6 University of Southampton, Southampton, UK; 7 NIHR Moorfields Clinical Research Facility, Moorfields Eye Hospital, London, UK; 8 Dept of Ophthalmology, Oxford Eye Hospital, Oxford University Hospitals NHS Trust, Oxford, UK; 9 Aswan University Hospital, Aswan, Egypt; 10 NIHR Biomedical Research Centre at Moorfields Eye Hospital NHS Foundation Trust and UCL Institute of Ophthalmology, London, UK; 11 Centre for Optometry & Vision Sciences, School of Biomedical Sciences, Ulster University, Coleraine, Northern Ireland UK; 12 Department of Medical and Surgical Specialties, Radiological Sciences, and Public Health, University of Brescia, Brescia, Italy; 13 Macular Society, Andover, UK; 14 Fight for Sight, London, UK; 15 Moorfields Eye Charity, Moorfields Eye Hospital, London, UK; 16 Eye care and living well services, Royal National Institute of Blind people, London, UK; 17 Bradford Teaching Hospitals, Royal Infirmary, Bradford, West Yorkshire UK; 18 York Hospital, University of York, York, UK; 19 Department of Ophthalmology, London North West University Healthcare NHS Trust, London, UK; 20 Brain Sciences, Imperial College London, London, UK; 21 NIHR Clinical Research Network, University of Liverpool, Liverpool, UK; 22 Maidstone & Tunbridge Wells NHS Trust, Institute of Medical Sciences, Canterbury Christ Church, University, Maidstone, UK; 23 Eye and Vision Science, University of Liverpool, Liverpool, UK; 24 CRN National Coordinating Centre, Leeds, UK; 25 Western Eye Hospital, Imperial College London & UCL Institute of Ophthalmology Imperial College Healthcare NHS Trust, London, UK; 26 Oxford University Hospitals NHS Foundation Trust, London, UK; 27 Nuffield Laboratory of Ophthalmology, Nuffield Department of Clinical Neuroscience University of Oxford, Oxford Eye Hospital, John Radcliffe, Oxford, UK; 28 Kings College London, London, UK; 29 Royal Victoria Hospital, Belfast, UK; 30 Nottingham University Hospital, Academic Ophthalmology, University of Nottingham Medical School, Nottingham, UK; 31 Faculty of Medicine, University of Southampton, Southampton, UK; 32 NIHR Clinical Research Network Coordinating Centre, University of Liverpool, Liverpool, UK; 33 Institute of Population Health, University of Liverpool, Liverpool, UK; 34 Gloucestershire Hospitals NHS Foundation Trust, Cheltenham, UK; 35 Somerset NHS Foundation Trust, Yeovil, UK; 36 Belfast Health & Social Care Trust, Belfast, UK; 37 Birmingham and Midland Eye Centre, Birmingham, UK; 38 Princess Alexandra Eye Pavilion and University of Edinburgh, Edinburgh, UK; 39 Manchester University NHS Foundation Trust and University of Manchester, Manchester, UK; 40 Sunderland Eye Infirmary, South Tyneside and Sunderland NHS Foundation Trust, London, UK; 41 University Hospital Wales and Cardiff University, London, UK; 42 The College of Optometrists, London, UK; 43 Royal Marsden NHS Foundation Trust, London, UK; 44 Blind Veterans, London, UK; 45 Moorfields Eye Hospital, London, UK; 46 NIHR Trainee Research Network Representative (Northern region), London, UK; 47 BIOS Research The University of Reading, Reading, UK; 48 Retina, London, UK; 49 NIHR Trainee Research Network Representative (Southern region), London, UK

**Keywords:** Outcomes research, Education

## Abstract

**Objectives:**

To validate and update the 2013 James Lind Alliance (JLA) Sight Loss and Vision Priority Setting Partnership (PSP)’s research priorities for Ophthalmology, as part of the UK Clinical Eye Research Strategy.

**Methods:**

Twelve ophthalmology research themes were identified from the JLA report. They were allocated to five Clinical Study Groups of diverse stakeholders who reviewed the top 10 research priorities for each theme. Using an online survey (April 2021-February 2023), respondents were invited to complete one or more of nine subspecialty surveys. Respondents indicated which of the research questions they considered important and subsequently ranked them.

**Results:**

In total, 2240 people responded to the survey (mean age, 59.3 years), from across the UK. 68.1% were female. 68.2% were patients, 22.3% healthcare professionals or vision researchers, 7.1% carers, and 2.1% were charity support workers. Highest ranked questions by subspecialty: Cataract (prevention), Cornea (improving microbial keratitis treatment), Optometric (impact of integration of ophthalmic primary and secondary care via community optometric care pathways), Refractive (factors influencing development and/or progression of refractive error), Childhood onset (improving early detection of visual disorders), Glaucoma (effective and improved treatments), Neuro-ophthalmology (improvements in prevention, diagnosis and treatment of neurodegeneration affecting vision), Retina (improving prevention, diagnosis and treatment of dry age-related macular degeneration), Uveitis (effective treatments for ocular and orbital inflammatory diseases).

**Conclusions:**

A decade after the initial PSP, the results refocus the most important research questions for each subspecialty, and prime targeted research proposals within Ophthalmology, a chronically underfunded specialty given the substantial burden of disability caused by eye disease.

## Introduction

Ophthalmology is a rapidly growing research area in the UK recruiting on average more than 15,000 patients into clinical research trials annually with most National Health Service (NHS) trusts participating in eye research [1]. Ophthalmology is one of the leading areas of novel treatments [1] and in the past few decades we have seen the introduction of novel diagnostic and treatment modalities that have markedly improved outcomes in people with eye diseases. Despite active research within ophthalmology, there are still unanswered questions about prevention, diagnosis, and treatment of eye conditions and sight loss, half of which is presumed avoidable (although the UK lacks nationally-representative population-based prevalence data) [2]. Funding for eye research is limited [3], so it is important to identify the unanswered questions of highest clinical importance so that research targeting greatest needs can be well invested for the benefits of patients and public in the future [4].

The UK Vision Strategy was developed in 2008 to set the framework to address the issues on visual impairment in society and research was identified as one of the important strategies [4]. The Vision 2020 UK Eye Research Group was formed subsequently as part of the Vision 2020 initiative to minimise avoidable visual impairment as well as to reduce the impact of unavoidable sight loss [5]. It aimed to set priorities for the research agendas using well-constructed methods by collaboration with the James Lind Alliance (JLA) [4]. JLA is a non-profit organisation that has been working in partnership with stakeholders including patients, their representatives, and clinicians to set research priorities in a wide range of conditions since 2004. The JLA Priority Setting Partnerships (PSPs) reflect the views of current NHS service users and clinicians to prioritise funding for research that is of high clinical relevance.

The Sight Loss and Vision PSP was formed in 2012 to launch a project for eye research priority setting in collaboration with the JLA [4]. This was overseen by a steering committee made up of diverse backgrounds of patients, clinicians, and the representatives from sight loss organisations and the project was funded by the College of Optometrists, Fight for Sight, National Institute for Health and Care Research (NIHR) Moorfields Biomedical Research Centre, Royal National Institute of Blind People, Royal College of Ophthalmologists, and UK Vision Strategy. In 2013, their Sight Loss and Vision Loss Report published top 10 lists of research priorities across 12 ophthalmology subspecialities following surveys and consultations with more than 2000 ophthalmology stakeholders, supported by the JLA [4]. This was the first time in the world that research priorities were set in ophthalmology based on the systematic approach of reaching consensus from service users and providers [5].

However, there is still room for more patient-centred research, especially in those subspecialties that carry high clinical burden in the NHS [1]. There is also a growing role of commercial studies in the UK which may affect the paradigm of future eye research in the UK [1]. In addition, there have been emerging new eye treatments available in the NHS over the last decades and new models of care designed to make the services more efficient. The Covid-19 pandemic has further introduced some changes to our clinical practice and treatment goals. As such, it is time to revisit the original research priorities to ensure they still reflect current health needs.

The NIHR Clinical Research Network (CRN; which will become the NIHR Research Delivery Network in late 2024) has supported a transformation in the strength of England’s research delivery system, promoted the successful delivery of studies and underpinned the dramatic expansion of health research participation. The CRN’s Ophthalmology Specialty Group represents Ophthalmology within this network and oversees clinical research into medical and surgical treatments of eye diseases, optometry, visual rehabilitation and other key areas within the broader discipline of vision sciences [6]. The CRN’s Ophthalmology Specialty Group initiated a UK Clinical Eye Research Strategy in 2020 [7]. This strategy started with a major initiative to update the previous James Lind Alliance (JLA) Sight and Vision Loss Priority Setting Partnership (PSP) [4]. The results are the subject of this report.

## Methods

In January 2021, a meeting was convened with the original JLA facilitator involved in 2013 to look at exploring a suitable methodology to validate and update the research priorities already identified from this report. We sought a pragmatic and novel approach as an updated PSP methodology did not exist at that time. A two-phase process was agreed.

Phase 1: Twelve ophthalmology research themes/sub-specialties identified from the 2013 Sight and Vison Loss JLA report [4] were allocated to five Clinical Study Groups (CSGs), which cover 9 different subspecialties. The formation of CSGs is an initial output of the UK Clinical Eye Research Strategy, and each is chaired by an ophthalmologist with a strong clinical research record (GS, AAB, JS, SS, SS). Each Chair was tasked with convening a committee of diverse stakeholders to collectively review the top 10 research priorities of each of these themes. The consensus from the five CSG areas was that the majority of JLA research priority questions remained valid as they were generally very broad in scope. When considered appropriate, CSG committees refined some original questions and added others.

Phase 2: An online survey was designed using SurveyMonkey software (SurveyMonkey Inc., San Mateo, California, USA; the online survey’s landing page is shown in Appendix 1). The survey went live on 19 April 2021 and closed in February 2023. A formal communication and dissemination strategy (Appendix 2) was agreed with the NIHR which included distribution of the survey link to a wide range of organisations across the UK including the Ophthalmology Specialty Group leads for each region of England and devolved nations. A direct contact person for each organization was established (usually the communications lead) to promote and disseminate the survey. In advance of this, the NIHR worked with the project team to produce a press release with a direct link to the survey. An NIHR press release had a Quick Response (QR) code added as another method to direct respondents to the online survey. Each organization sent the survey to all of its members as well as adding the links on to e-newsletters, direct emails to their members with the survey links above and promoting via their own organization websites and via Twitter (a social media website, now known as X).

National and local charities and professional organisations were involved in its dissemination strategy which aimed to maximise diversity, for example among minority ethnic groups and across all four devolved nations.

Respondents were able to enter anonymized data, in response to questions regarding demographics of the respondents, which included questions regarding age, sex, ethnicity, nation within the UK, and whether respondents were health care professionals, vision researchers, charity support workers or patients and carers. One or more of the nine surveys could be selected for completion by respondents from the following subspecialties: cataract, cornea, childhood-onset disorders, glaucoma, neuro-ophthalmology, optometry, refractive disorders, retinal disease, and uveitis.

Within each of these subspecialties, there were two tasks. The first task presented at least 10 research questions and requested the respondent to indicate which they felt was important. Each research question was also accompanied by a lay explanation. The second task listed only the research questions which the respondent had indicated were important and requested the respondent to rank these questions in a scale 1–10. A freetext option was optional for respondents to add comments. Descriptive statistics were used for the analysis involving frequency distribution, central tendency, and variability of the data set.

## Results

**Fig. 1:**
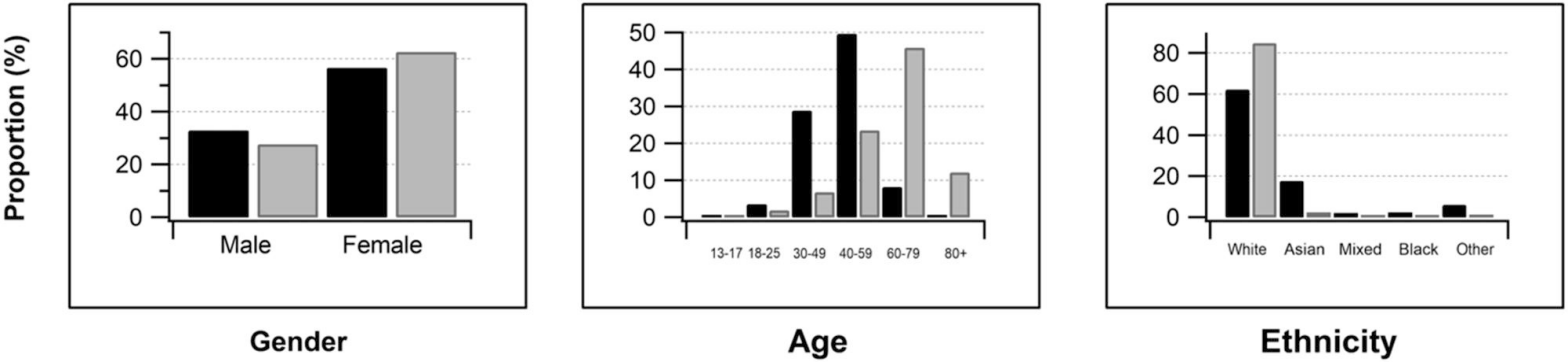
Proportion of healthcare practitioners (black bars) and non-healthcare practitioner respondents (grey bars) by gender, age and ethnicity.

A total of 2240 people responded to the survey, with an average age of 59.3 years; 87.9% were from England, 5.5% from Scotland, 4.4% from Wales, and 2.2% from Northern Ireland. Of those respondents who gave their sex, 704 (31.9%) were male and 1501 (68.1%) female. 1527 (68.2%) respondents were patients, 158 (7.1%) carers, 499 (22.3%) healthcare professionals or vision researchers, and 48 (2.1%) charity support workers. In terms of ethnicity, 1959 (87.5%) respondents identified as white, 139 (6.2%) as Asian/ Asian British, 36 (1.6%) as Mixed/Multiple ethnic groups, and 31 (1.4%) as Black/African/Caribbean/Black British. Demographics of healthcare practitioners and non-healthcare practitioners are given in Fig. 1.

**Fig. 2:**
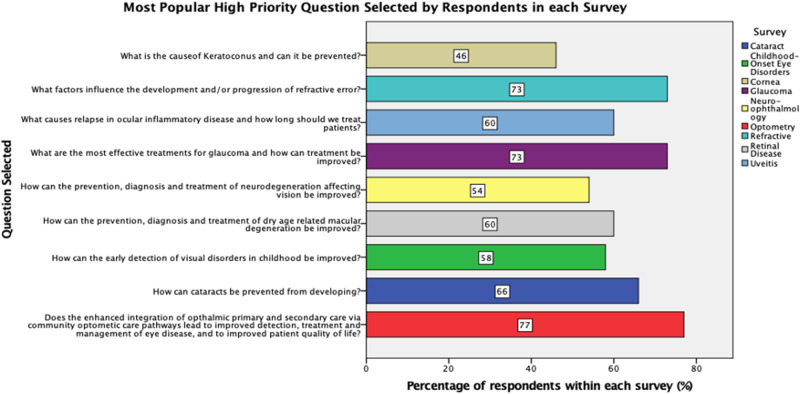
Most popular high priority question selected by respondents in each survey.

The research question most commonly ranked as of highest priority is given in Fig. 2 for each of the nine subspecialties. These and all top 10 research priorities for each of the 9 subspecialty surveys are presented in Tables 1–3.

**Table 1:** Top 10 research priorities for cataract, cornea, optometric, refractive topics.

**CATARACT 283 respondents**	**% of those responding to this survey who judged this research question to be important**	**Average ranking of this research question by respondents On a scale of 1–10 (1 most important, 10 least)**
Q1 How can cataracts be prevented from developing?	66.43% (188 respondents)	2.97
Q2 What is the cause of cataract? How do cataracts form?	45.23% (128 respondents)	3.14
Q3 How can cataract surgery outcomes be improved?	53.71% (152 respondents)	3.38
Q4 How can cataract progression be slowed down?	51.24% (145 respondents)	3.74
Q5 What alternatives to treat cataracts other than cataract surgery are being developed?	49.12% (139 respondents)	3.87
Q6 What is the best measure of visual disability due to cataract?	37.46% (106 respondents)	3.89
Q7 How safe is bilateral simultaneous surgery?	23.32% (66 respondents)	4.03
Q8 Can the return of cloudy or blurred vision after cataract surgery known as posterior capsule opacity (PCO) or secondary cataract be prevented?	60.78% (172 respondents)	4.09
Q9 Can retinal detachment be prevented after cataract surgery?	47.35% (134 respondents)	4.12
Q10 Should we be looking at developing or using certain emerging or existing technologies in cataract care?	32.51% (92 respondents)	4.40
Q 11 Should accommodative lenses be developed for cataract surgery?	44.17% (125 respondents)	4.45
Q12 What are the roles of telemedicine/remote medicine, AI, electronic patient records, smart theatres, OCT, biometry and other technologies in the future of cataract care?	24.03% (68 respondents)	4.84
Q13 What are the outcomes for cataract surgery among people with different levels of cognitive impairment (whatever the cause but including dementia, stroke, neurological conditions, head injuries)?	22.61% (64 respondents)	5.05
**CORNEA** **140 Respondents**		
Q1 How can microbial keratitis treatment be improved?	23.57% (33 respondents)	3.00
Q2 What is the cause of Keratoconus and can it be prevented?	46.43% (65 respondents)	3.11
Q3 How can we prevent Keratoconus progression?	40.71% (57 respondents)	3.26
Q4 How can dry eye treatment be improved?	46.43% (65 respondents)	3.53
Q5 How can quality of life of contact lenses wearer for Keratoconus disease be improved?	29.29% (41 respondents)	3.59
Q6 How can the rejection of corneal transplants be prevented?	43.57% (61 respondents)	3.80
Q7 How can diagnosis of corneal infections be improved and how can corneal infection be prevented in high-risk individuals?	28.57% (40 respondents)	3.81
Q8 How can detection of progression in Keratoconus patients be improved ?	35.00% (49 respondents)	3.86
Q9 How to standardize the diagnosis and monitoring of dry eye?	35.71% (50 respondents)	3.89
Q10 How can ocular surface disease in children, such as blepharokeratoconjunctivitis and vernal keratoconjunctivitis be managed better?	17.86% (25 respondents)	3.91
Q11 How can utilization of corneal donor tissues be improved?	33.57% (47 respondents)	3.98
Q12 How can non-surgical therapy for Corneal endothelial dysfunctions be developed?	32.14% (45 respondents)	4.00
Q13 How can ocular complications associated with Stevens Johnson Syndrome be improved?	10.71% (15 respondents)	4.07
Q14 How can visual outcomes of corneal transplantation be improved?	46.43% (65 respondents)	4.52
Q15 How can telemedicine be improved for diagnosis, management and treatment of ocular surface disease ?	16.43% (23 respondents)	5.10
Q16 How can corneal transplant complication related to vaccinations be improved ?	11.43% (16 respondents)	5.33
**OPTOMETRIC** **194 Respondents**		
Q1 Does the enhanced integration of ophthalmic primary and secondary care via community optometric care pathways lead to improved detection, treatment and management of eye disease, and to improved patient quality of life?	77.32% (150 respondents)	1.67
Q2 How can novel medical devices and technology be applied to improve the prevention, diagnosis, and management of eye disease?	66.49% (129 respondents)	1.98
Q3 What are the most appropriate measures of visual function, structure and vision-related quality-of-life for the detection and monitoring of cataracts?	51.55% (100 respondents)	2.34
Q4 How can the detection, diagnosis and management of ocular surface disorders be improved?	41.24% (80 respondents)	2.71
Q5 Can corneal infections be prevented in high-risk individuals such as contact lens wearers?	35.05% (68 respondents)	3.22
Q6 What is the most effective management of ocular complications associated with Stevens Johnson Syndrome?	15.46% (30 respondents)	4.14
**REFRACTIVE** **107 Respondents**		
Q1 What factors influence the development and/or progression of refractive error (short-sightedness, astigmatism, presbyopia and long-sightedness?)	72.90% (78 respondents)	2.20
Q2 How does the wearing of spectacles (of any prescription) affect the progression of refractive error?	61.68% (66 respondents)	3.12
Q3 No intraocular lens provides as good vision and range of vision as the natural human crystalline lens, how can intraocular lens implants be further improved and their outcomes compared in a standardised way?	36.45% (39 respondents)	3.26
Q4 Could the accurate testing of refractive error be made less dependent on a subjective response ie. the person’s own response?	48.60% (52 respondents)	3.52
Q5 What are the economic and social burdens of refractive error?	53.27% (57 respondents)	3.45
Q6 Could the accurate testing of refractive error be made less dependent on a subjective response ie. the person’s own response?	48.60% (52 respondents)	3.52
Q7 To develop new treatments for presbyopia?	32.71% (35 respondents)	4.13
Q8 What factors influence the development and/ or progression of Keratoconus?	28.04% (30 respondents)	4.13
Q9 What are the long term outcomes of refractive surgery?	33.64% (36 respondents)	4.36
Q10 Are there any alternatives or better treatments for Keratoconus other than corneal collagen cross-linking?	24.30% (26 respondents)	4.40
Q11 There are many types of laser vision correction, does one have better long term outcomes and less risk of complications?	26.17% (28 respondents)	4.41
Q12 Can dry eye after laser vision correction be better treated or prevented?	14.95% (16 respondents)	4.65
Q13 What is the best way to quantify quality of vision objectively before and after refractive surgery?	28.04% (30 respondents)	4.74
Q14 How can biometry (measurement of ocular structures) and selection of the required intraocular lens implant (lens power calculations) be improved?	23.36% (25 respondents)	4.75
Q15 What are the risk factors for corneal ectasia (warping or bulging of the corneal shape) after laser vision correction and when does a cornea become at risk of ectasia following laser vision correction?	12.15% (13 respondents)	6.08

**Table 2:** Top 10 research priorities for childhood onset*, glaucoma, and neuro-ophthalmology topics.

**CHILDHOOD ONSET 177 respondents**	**% of those responding to this survey who judged this research question to be important**	**Average ranking of this research question by respondents On a scale of 1–10 (1 most important, 10 least)**
Q1 How can the early detection of visual disorders in childhood be improved?	58.19% (103 respondents)	3.43
Q2 What improvements can be made in the assessment of visual function in children, including outcome measures for clinical studies and vision-related quality of life?	47.46% (84 respondents)	3.51
Q3 How can the prevention, diagnosis and treatment of Cerebral Visual Impairment (CVI) in children be improved?	38.98% (69 respondents)	3.52
Q4 How can the prevention, diagnosis and treatment of nystagmus and albinism be improved?	41.24% (73 respondents)	3.64
Q5 How can the prevention, diagnosis and treatment of refractive error in children be improved?	37.85% (67 respondents)	3.82
Q6 How can the prevention, diagnosis and treatment of amblyopia (Lazy eye) be improved?	38.98% (69 respondents)	4.01
Q7 How can genomic medicine be exploited to improve the prevention, diagnosis and treatment of childhood disorders of vision?	41.24% (73 respondents)	4.01
Q8 How can the diagnosis and treatment of inherited retinal disorders be improved?	40.68% (72 respondents)	4.13
Q9 How can the prevention, diagnosis and treatment of ocular, orbital and visual pathway tumours in children be improved?	31.07% (55 respondents)	4.43
Q10 How can the prevention, diagnosis and treatment of visual loss caused by prematurity be improved?	32.77% (58 respondents)	4.45
Q 11 How can ‘best practice’ be standardised for children with rare visual disorders?	38.42% (68 respondents)	4.57
Q12 How can biomarkers and bioresources be exploited to improve the prevention, diagnosis and treatment of childhood disorders of vision?	28.81% (51 respondents)	4.67
Q13 How can the prevention, diagnosis and treatment of Strabismus be improved?	32.20% (57 respondents)	4.71
Q14 How can the diagnosis and treatment of childhood cataracts be improved?	19.77% (35 respondents)	4.88
Q15 How can the prevention, diagnosis and treatment of optic nerve disorders, including glaucoma, in children be improved?	33.33% (59 respondents)	5.41
**GLAUCOMA** **651 Respondents**		
Q1 What are the most effective treatments for glaucoma and how can treatment be improved?	72.96% (475 respondents)	2.11
Q2 How can any vision loss be restored for people with glaucoma?	65.75% (428 respondents)	2.44
Q3 What can be done to avoid late diagnosis of sight-threatening glaucoma?	54.84% (357 respondents)	2.48
Q4 What is the most effective way of monitoring the progression of glaucoma?	64.36% (419 respondents)	2.70
Q5 What causes glaucoma?	53.00% (345 respondents)	2.71
Q6 Is there a link between treatment adherence and glaucoma progression and how can adherence be improved?	32.10% (209 respondents)	3.88
**NEURO-OPHTHALMOLOGY** **253 Respondents**		
Q1 How can the prevention, diagnosis and treatment of neurodegeneration affecting vision be improved?	54.15% (137 respondents)	2.84
Q2 How can biomarkers and bio-resources be exploited to improve the prevention, diagnosis, monitoring and treatment of adult neuro-ophthalmic disorders?	45.06% (114 respondents)	2.96
Q3 How can the prevention, diagnosis and treatment of acquired optic neuropathies be improved?	43.87% (111 respondents)	2.96
Q4 How can the prevention, diagnosis and treatment of neuroinflammation affecting vision be improved?	42.29% (107 respondents)	3.09
Q 5 How can the prevention, diagnosis and treatment of stroke affecting vision be improved?	46.64% (118 respondents)	3.12
Q 6 How can the prevention, diagnosis and treatment of intracranial tumours affecting vision be improved?	34.39% (87 respondents)	3.16
Q 7 How can the prevention, diagnosis and treatment of hereditary optic neuropathies be improved?	44.66% (113 respondents)	3.36
Q 8 How can the diagnosis and treatment of traumatic brain injury (TBI) affecting vision be improved?	39.53% (100 respondents)	3.72
Q 9 How can the prevention, diagnosis and treatment of strabismus in adults be improved?	29.64% (75 respondents)	3.89

**Table 3:** Top 10 research priorities for retina and uveitis topics.

**RETINA 595 Respondents**	**% of those responding to this survey who judged this research question to be important**	**Average ranking of this research question by respondents On a scale of 1–10 (1 most important, 10 least)**
Q1 How can the prevention, diagnosis and treatment of dry age-related macular degeneration be improved?	59.83% (356 respondents)	2.44
Q2 How can the prevention, diagnosis and treatment of wet age related macular degeneration be improved?	55.80% (332 respondents)	2.49
Q3 How can sight loss due to inherited retinal diseases be prevented or restored?	56.64% (337 respondents)	2.54
Q4 Visual rehabilitation in eyes with central visual loss due to retinal diseases?	49.41% (294 respondents)	3.20
Q5 How can the prevention, diagnosis and treatment of diabetic eye disease be improved?	34.45% (205 respondents)	3.25
Q6 Artificial Intelligence in retinal diseases	34.62% (206 respondents)	3.61
Q7 How can the prevention, diagnosis and treatment of macular holes be improved?	36.47% (217 respondents)	3.63
Q8 How can the prevention, diagnosis and treatment of fibrosis as a complication of retinal diseases be improved?	20.67% (123 respondents)	4.06
Q9 How can the prevention, diagnosis and treatment of ocular inflammatory disease be improved?	21.51% (128 respondents)	4.21
Q10 How can the prevention, diagnosis and treatment of ocular melanoma be improved? - Cancer can occur in the eye first or affect the eye from other parts of the body.	15.80% (94 respondents)	5.12
**UVEITIS** **151 Respondents**		
Q1 What are the most effective treatments for ocular and orbital inflammatory diseases?	53.64% (81 respondents)	2.62
Q2 What causes relapse in ocular inflammatory disease and how long should we treat patients?	59.60% (90 respondents)	2.64
Q3 What are the best ways to personalise treatment in uveitis and scleritis?	53.64% (81 respondents)	2.88
Q4 What causes uveitis or scleritis in isolated ocular disease and in systemic disease with associated disease?	55.63% (84 respondents)	2.99
Q5 Which licensed treatments for systemic inflammatory diseases (but not for uveitis) are effective in inflammatory eye disease?	42.38% (64 respondents)	3.12
Q6 What are the most effective biomarkers (imaging / non-imaging) to predict relapse or monitor for disease progression in ocular or orbital inflammatory disease?	48.34% (73 respondents)	3.21
Q7 How can we improve ways to diagnose infectious uveitis?	33.77% (51 respondents)	3.55
Q8 What are the most effective scoring systems and clinical outcome measures (imaging / non-imaging) of disease and treatment response in ocular or orbital inflammatory disease?	33.11% (50 respondents)	3.70
Q9 What is the cause and most effective medical management for Thyroid Eye Disease?	18.54% (28 respondents)	4.52

Highest ranked questions by subspecialty can be summarized as follows: cataract (prevention), cornea (improving microbial keratitis treatment), optometric (impact of integration of ophthalmic primary and secondary care via community optometric care pathways), refractive (factors influencing development and/or progression of refractive error), childhood onset (improving early detection of visual disorders), glaucoma (effective and improved treatments), neuro-ophthalmology (improvements in prevention, diagnosis and treatment of neurodegeneration affecting vision), retina (improving prevention, diagnosis and treatment of dry age-related macular degeneration), uveitis (effective treatments for ocular and orbital inflammatory diseases). Understanding the cause and most effective medical management for thyroid eye disease was also highlighted as an important research priority.

## Discussion

Priority Setting Partnerships enable clinicians, patients and carers to work together to identify and prioritise evidence-based uncertainties in particular areas of health and care that could be answered by research. This informs researchers and research funders about priorities so that they can tailor their research making it as meaningful as possible and targeted to those people who most need it while making a wider impact. We have described an iterative process that first established wide stakeholder engagement within each of the subspecialties of Ophthalmology, then reviewed and refined the original 2013 JLA outputs of the PSP [4], and finally disseminated these research questions to a large group of 2240 respondents. The results provide a ‘refresh’ of the most important research questions for each of these subspecialties of Ophthalmology a decade after the initial PSP.

Comparing this latest work with that of the original PSP, the residence of respondents was very similar, differing by less than 1 percentage point for each of the nations (original PSP respondents: England 89%, Scotland 6%, Wales 4%, Northern Ireland 1%). Average age of the original PSP was 65.7 years which was slightly higher than the average age of 59.3 years in this recent survey. The sex of respondents was relatively similar to the original PSP (males 38%, females 62%) and the proportion of healthcare professional respondents (16% in the original PSP). The total number of respondents was also similar (2220 participated in the original PSP). Ethnicity of respondents was not reported in the original PSP.

Within the refractive error, cataract and glaucoma subspecialties, the top priority research question remained the same in both the original PSP and this recent survey, yet there was variance among other subspecialties in terms of highest ranked questions. The design of the original PSP and this most recent survey is of course different involving different respondents, and the purpose of the recent survey was not to repeat the original PSP but rather to provide an up-to-date perspective on what a wide group of stakeholders judge as most important.

Strengths of this study include the large and diverse stakeholder group involved in each of the topic areas in Phase 1 and the large sample that answered the online survey in Phase 2. Additionally, ranking of the research questions in order of importance offers some insight into their relative importance from the perspective of the respondents. Although significant efforts were made to disseminate the survey to as diverse a group as possible, the representativeness of the sample among the patient population is unknown. For example, socio-economically deprived populations may not be well represented on account of online access and other factors. An additional limitation was the low proportion of non-white respondents. It should also be noted that thyroid eye disease was included among the uveitis section for convenience, yet it is not a uveitic condition.

The next step to be taken by the UK Clinical Eye Research Strategy will be to take the highest priority research questions, and within subspecialties, work up research proposals around these using a Patient/Population/Problem, Intervention/exposure, Comparison/Control, and Outcome measure (PICO) methodology [8, 9]. This is a pressing issue as more commissioned calls for clinical Ophthalmology research will increase the amount of research funding directed towards Ophthalmology which currently receives significantly less NIHR research grant funding than other medical specialties. This imbalance is a particular concern given the substantial burden of disability caused by eye disease, which in terms of disability-adjusted life-years, is not commensurate with the funding received.

## Summary

### What was known before

- The Sight Loss and Vision Priority Setting Partnership was formed in 2012 to launch a project for eye research priority setting in collaboration with the James Lind Alliance (JLA).
- In 2013, the Sight Loss and Vision Loss Report published top 10 lists of research priorities across 12 ophthalmology subspecialities following surveys and consultations with more than 2000 ophthalmology stakeholders, supported by the JLA.
- This was the first time in the world that the research priority was set in ophthalmology based on the systematic approach on reaching consensus from the service users and providers.

### What this study adds

- The NIHR Clinical Research Network’s Ophthalmology Specialty Group initiated a UK Clinical Eye Research Strategy in 2020.This strategy started with a major initiative to update the previous James Lind Alliance (JLA) Sight Loss and Vision Priority Setting Partnership.
- 2240 persons responded to a recent online survey that invited respondents to complete one or more of nine subspecialty surveys.
- Respondents indicated which of the research questions they considered important and subsequently ranked them.
- Highest ranked questions by subspecialty: Cataract (prevention), Cornea (improving microbial keratitis treatment), Optometric (impact of integration of ophthalmic primary and secondary care via community optometric care pathways), Refractive (factors influencing development and/or progression of refractive error), Childhood onset (improving early detection of visual disorders), Glaucoma (effective and improved treatments), Neuro-ophthalmology (improvements in prevention, diagnosis and treatment of neurodegeneration affecting vision), Retina (improving prevention, diagnosis and treatment of dry age-related macular degeneration), Uveitis (effective treatments for ocular and orbital inflammatory diseases) A decade after the initial PSP, the results refocus the most important research questions for each subspecialty, and prime targeted research proposals within Ophthalmology.

### Supplementary information

Appendix 1- Online Survey

Appendix 2- Dissemination Strategy

Appendix 3 - Contributions by Authors

## Data Availability

The data that support the findings of this study are available from the Chair of the UK Clinical Eye Research Strategy, Professor Rupert Bourne; rb@rupertbourne.co.uk upon reasonable request. Data are located in controlled access data storage at Anglia Ruskin University, Cambridge, UK.
