# Supplementary material for: The UK clinical eye research strategy: refreshing research priorities for clinical eye research in the UK
Source: Eye (Lond). 2024 May 29;38(10):1947–57. doi: 10.1038/s41433-024-03049-6 (PMC11226710; doi:10.1038/s41433-024-03049-6)
Supplement: Supplementary file 1 — Appendix 1- Online Survey [file 41433_2024_3049_MOESM1_ESM.pdf]

Eye Research Priority Survey

Invitation to Participate

An eye research priority setting exercise is being undertaken and initiated by the NIHR Ophthalmology Specialty Group as part of the UK Clinical Eye Research Strategy. The aim of this priority setting exercise is to update the hugely useful James Lind Alliance (JLA) Priority Setting Partnership (PSP) for Sight Loss and Vision 2013 and help guide eye research in the future. We invite anyone working within the eye care sector or cared for by the health service or supported by charities to let us know their thoughts about which of the research areas should be prioritised, by completing a simple 2-page short survey online. Your contributions will inform eye research for years to come. Depending on your areas of interest, you can complete one or more of the 9 topic-specific surveys, and this should only take a few minutes.

The Royal College of Ophthalmologists and the College of Optometrists are delighted to support this initiative along with other stakeholders.

How we will store and use your information

We do not require your contact details in this survey, but you may provide them on a voluntary basis. Your details will be used to contact you if you wish to take part in the next step of the process (framing research priorities into research questions leading to appropriate study designs that initiate new research projects). We will keep your information secure according to data protection legislation in your relevant jurisdiction. If you would like to contact us regarding any concerns or further information please email us at: contact@ukeyeresearchstrategy.ac.uk

* I confirm that I understand the above and consent to take part in this survey

- ◯ Yes
- ◯ No

Eye Research Priority Survey

* Which of the following best describes you?

- ◯ A patient
- ◯ A carer for a patient
- ◯ A healthcare professional and/or vision researcher
- ◯ A charity support worker
- ◯ Other

Eye Research Priority Survey

* As you clicked on "**other**" in the previous question, please elaborate further in free text box below.

Eye Research Priority Survey

*** Please select the type of professional you are**

- ◯ Ophthalmologist
- ◯ Optometrist
- ◯ Orthoptist
- ◯ Other
- ◯ Nurse

Eye Research Priority Survey

As you clicked on **"other"** in the previous question, please elaborate further in free text box below.

* Please describe the type of professional you are

Eye Research Priority Survey

*** Which of the following best describes your role?**

- ◯ an academic with an interest in vision research
- ◯ a clinical role **without** research involvement
- ◯ a clinical role **with** research involvement
- ◯ Other

Eye Research Priority Survey

As you clicked on **"other"** in the previous question, please elaborate further in free text box below.

* Please elaborate further regarding your role

Eye Research Priority Survey

*** Where do you live in the UK?**

- ◯ England
- ◯ Wales
- ◯ Northern Ireland
- ◯ Scotland

Eye Research Priority Survey

*** What is your age?**

- ◯ 8-12
- ◯ 13-17
- ◯ 18-25
- ◯ 26-39
- ◯ 40-59
- ◯ 60-79
- ◯ 80+
- ◯ I prefer not to say

Eye Research Priority Survey

*** What is your gender?**

◯ Male

◯ Female

◯ Other

◯ Prefer not to say

Eye Research Priority Survey

* What is your ethnic group?

- ◯ White
- ◯ Mixed/Multiple ethnic groups
- ◯ Asian/Asian British
- ◯ Black/ African/Caribbean/Black British
- ◯ Other ethnic group

Moorfields Eye Charity | Macular Society | FIGHT FOR SIGHT | CERS UK Clinical Eye Research Strategy | NIHR National Institute for Health and Care Research

Eye Research Priority Survey

Survey Selection

Each survey is two pages long and should only take a few minutes to complete. Respondents are invited to complete one or more of these nine surveys that cover the following topics of eye research.

Definitions:

Neuro-ophthalmology: *Diseases involving the* **eye and the brain.**

Cataract: *Diseases involving the* **lens** *of the eye.*

Optometry: *Research involving* **optometrists (opticians)**, *the healthcare professionals concerned especially with examining the eye for defects especially* **refractive errors (short and long-sightedness)**, *their* **correction** *(including with* **glasses** *or* **contact lenses**), *and* **referring patients in** *for treatment.*

Cornea: *Diseases involving the* **front window of the eye**.

Retinal Disease: *Diseases involving the* **retina** *at the back of the eye, and the central retina area, the* **macula**, *where the central vision is damaged.*

Refractive: *Refractive error occurs when the eye cannot clearly focus the images from the outside world. The result* of **refractive errors** *(short and long-sightedness and astigmatism) is* **blurred vision**, *which is sometimes so severe that it causes visual impairment. These can be corrected with glasses, contact lenses or other potential methods.*

Uveitis: **Inflammation** *of the eye.*

Glaucoma: *A disease of the* **optic nerve** *which connects the eye to the brain, associated* **pressure** *in the eye and damage to the* **visual field** *if not detected early.*

Childhood-Onset Eye Disorders: *A disease of the* **eye** *and/or* **surrounding tissues**, *which may be* **isolated** *or as part of a* ***generalised medical condition***, *presenting in* **childhood**.

*** Please select the first survey you wish to complete**

- ◯ Neuro-ophthalmology
- ◯ Cataract
- ◯ Optometry
- ◯ Cornea
- ◯ Retinal Disease
- ◯ Childhood-Onset Eye Disorders
- ◯ Refractive
- ◯ Uveitis
- ◯ Glaucoma
- ◯ End Survey

Eye Research Priority Survey

Refractive Research: What should the research priorities be?

An eye research priority setting exercise being undertaken through the UK Clinical Eye Research Strategy. The aim of this priority setting exercise is to update the hugely useful James Lind Alliance (JLA) Priority Setting Partnership (PSP) for Sight Loss and Vision exercise of 2013 and help guide eye research in the future.

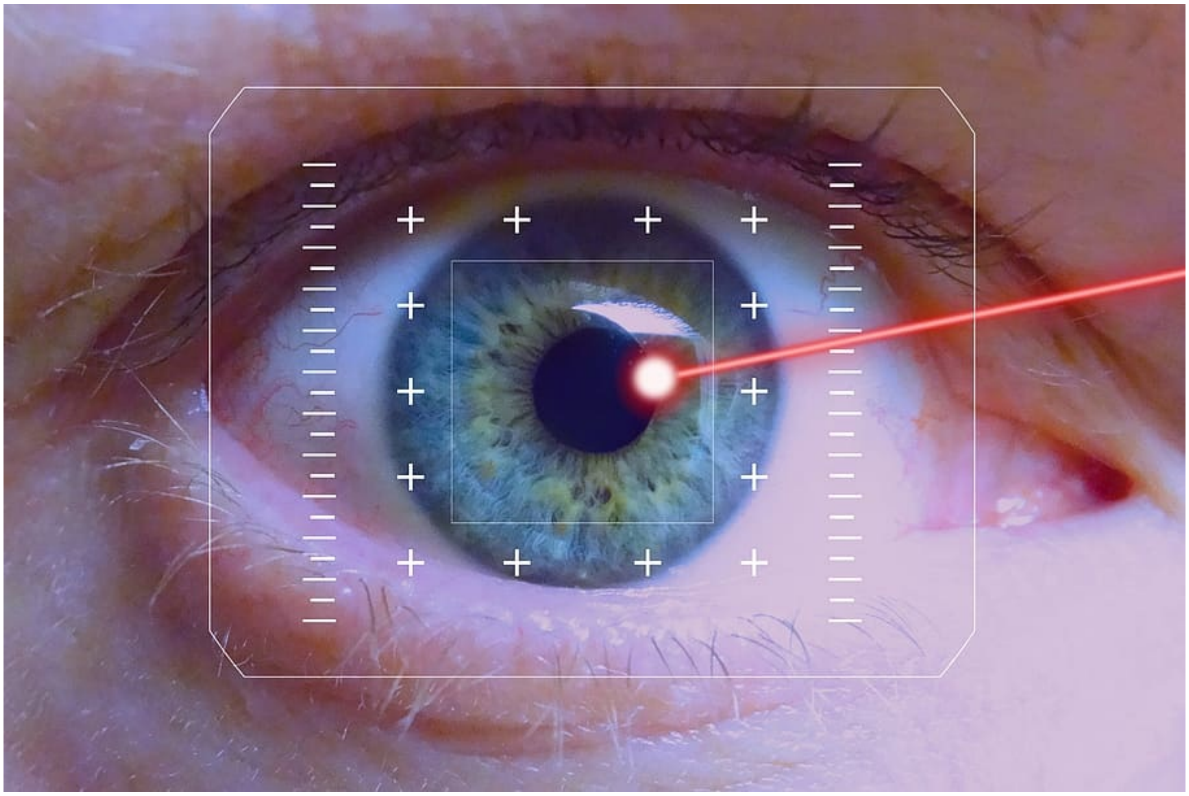

* Continue

◯ Yes

◯ No

Eye Research Priority Survey

Refractive Research Priorities

In this section, we are asking you to indicate which research priority questions you feel are important within Refractive Research. You will then be asked to rank each research question selected in order of priority.

* **Please select each research question you feel is a high priority up to a maximum of ten questions.**

- [ ] **What factors influence the development and/or progression of refractive error (short-sightedness, astigmatism, presbyopia and long-sightedness?** **-** *Identification of risk factors related to refractive error may lead to a better understanding of the natural progression and identification of potential new treatments.*

- [ ] **What are the economic and social burdens of refractive error?** - *Refractive error is a leading cause of visual impairment with high social and economic costs to treat.*

- [ ] **To develop new treatments for presbyopia** - *There is no treatment yet developed to prevent presbyopia and only limited options to currently treat presbyopia.*

- [ ] **What are the long term outcomes of refractive surgery?** - *There is limited data on the very long term outcomes (10 years+) of refractive surgery such as LASIK, PRK, SMILE and ICL implantation.*

- [ ] **Are there any alternatives or better treatments for Keratoconus other than corneal collagen cross-linking?** - *Corneal collagen crosslinking has revolutionised the treatment of Keratoconus, however, alternatives such as the development of eye drops would have better patient acceptability and potentially allow treatment at a much earlier stage.*

- [ ] **Can dry eye after laser vision correction be better treated or prevented?** - *Dry eye is common after laser vision correction and a significant patient concern/ issue.*

- [ ] **How can biometry (measurement of ocular structures) and selection of the required intraocular lens implant (lens power calculations) be improved?** - *The power of lens implants to attain the desired refractive result are selected by complex calculations based on various measurements of eye structures. Despite modern advancements including artificial intelligence, the desired outcome is still not achieved in some cases.*

- [ ] **There are many types of laser vision correction, does one have better long term outcomes and less risk of complications?** - *There are a number of different methods of laser vision correction – LASIK, PRK and SMILE, and there is limited data on the advantages/ disadvantages relative to each other.*

- [ ] **What are the risk factors for corneal ectasia (warping or bulging of the corneal shape) after laser vision correction and when does a cornea become at risk of ectasia following laser vision correction?** - *Although many risk factors for corneal ectasia after laser vision correction have been identified, it can still occur in the absence of these and it is unclear at what threshold a cornea becomes at significant risk of ectasia.*

- [ ] **What factors influence the development and/ or progression of Keratoconus? (natural warping or bulging of the corneal shape in the absence of previous corneal surgery)** - *Although many risk factors for Keratoconus have been identified, there are likely many novel ones that have yet been identified and these may lead to new treatment options.*

- [ ] **No intraocular lens provides as good vision and range of vision as the natural human crystalline lens, how can intraocular lens implants be further improved and their outcomes compared in a standardised way?** - *Better lens implant technology would lead to better quality of vision and range of vision without glasses so improving patient quality of life and reducing dependence on glasses.*

- [ ] ***What is the best way to quantify quality of vision objectively before and after refractive surgery?*** - *Visual acuity is typically measured using high contrast vision charts, yet these do not adequately describe other visual phenomena such as glare or halos, or range of depth of focus.*

- [ ] **How does the wearing of spectacles (of any prescription) affect the progression of refractive error?** - *Little is known about the natural history of refractive error and if/how current treatments may influence this.*

- [ ] **Could the accurate testing of refractive error be made less dependent on a subjective response ie. the person's own response?** - *The gold standard of measurement of refractive error is still subjective refraction which requires good subject participation and is a timely test. A machine-based measure that is faster and more reproducible and more accurate could potentially replace this.*

Eye Research Priority Survey

Refractive Research Priorities

In this section, we are asking you to indicate which research priority questions you feel are important within Refractive Research. You will then be asked to rank each research question selected in order of priority.

*** Please could you now rank the high priority questions selected in the previous question in order of priority? The questions can be dragged and dropped or ranked with one being the highest and ten being the lowest.**

What factors influence the development and/or progression of refractive error (short-sightedness, astigmatism, presbyopia and long-sightedness? - *Identification of risk factors related to refractive error may lead to a better understanding of the natural progression and identification of potential new treatments.*

What are the economic and social burdens of refractive error? - *Refractive error is a leading cause of visual impairment with high social and economic costs to treat.*

To develop new treatments for presbyopia - *There is no treatment yet developed to prevent presbyopia and only limited options to currently treat presbyopia.*

What are the long term outcomes of refractive surgery? - *There is limited data on the very long term outcomes (10 years+) of refractive surgery such as LASIK, PRK, SMILE and ICL implantation.*

There are many types of laser vision correction, does one have better long term outcomes and less risk of complications? - *There are a number of different methods of laser vision correction – LASIK, PRK and SMILE, and there is limited data on the advantages/ disadvantages relative to each other.*

What are the risk factors for corneal ectasia (warping or bulging of the corneal shape) after laser vision correction and when does a cornea become at risk of ectasia following laser vision correction? - *Although many risk factors for corneal ectasia after laser vision correction have been identified, it can still occur in the absence of these and it is unclear at what threshold a cornea becomes at significant risk of ectasia.*

What factors influence the development and/ or progression of Keratoconus? (natural warping or bulging of the corneal shape in the absence of previous corneal surgery) - *Although many risk factors for Keratoconus have been identified, there are likely many novel ones that have yet been identified and these may lead to new treatment options.*

Are there any alternatives or better treatments for Keratoconus other than corneal collagen cross-linking? - *Corneal collagen crosslinking has revolutionised the treatment of Keratoconus, however, alternatives such as the development of eye drops would have better patient acceptability and potentially allow treatment at a much earlier stage.*

Can dry eye after laser vision correction be better treated or prevented? - *Dry eye is common after laser vision correction and a significant patient concern/ issue.*

How can biometry (measurement of ocular structures) and selection of the required intraocular lens implant (lens power calculations) be improved? - *The power of lens implants to attain the desired refractive result are selected by complex calculations based on various measurements of eye structures. Despite modern advancements including artificial intelligence, the desired outcome is still not achieved in some cases.*

No intraocular lens provides as good vision and range of vision as the natural human crystalline lens, how can intraocular lens implants be further improved and their outcomes compared in a standardised way? - *Better lens implant technology would lead to better quality of vision and range of vision without glasses so improving patient quality of life and reducing dependence on glasses.*

What is the best way to quantify quality of vision objectively before and after refractive surgery? - *Visual acuity is typically measured using high contrast vision charts, yet these do not adequately describe other visual phenomena such as glare or halos, or range of depth of focus.*

How does the wearing of spectacles (of any prescription) affect the progression of refractive error? - *Little is known about the natural history of refractive error and if/how current treatments may influence this.*

Could the accurate testing of refractive error be made less dependent on a subjective response ie. the person’s own response? - *The gold standard of measurement of refractive error is still subjective refraction which requires good subject participation and is a timely test. A machine-based measure that is faster and more reproducible and more accurate could potentially replace this.*

Eye Research Priority Survey

Optometry Research: What should the research priorities be?

An eye research priority setting exercise being undertaken through the UK Clinical Eye Research Strategy. The aim of this priority setting exercise is to update the hugely useful James Lind Alliance (JLA) Priority Setting Partnership (PSP) for Sight Loss and Vision exercise of 2013 and help guide eye research in the future.

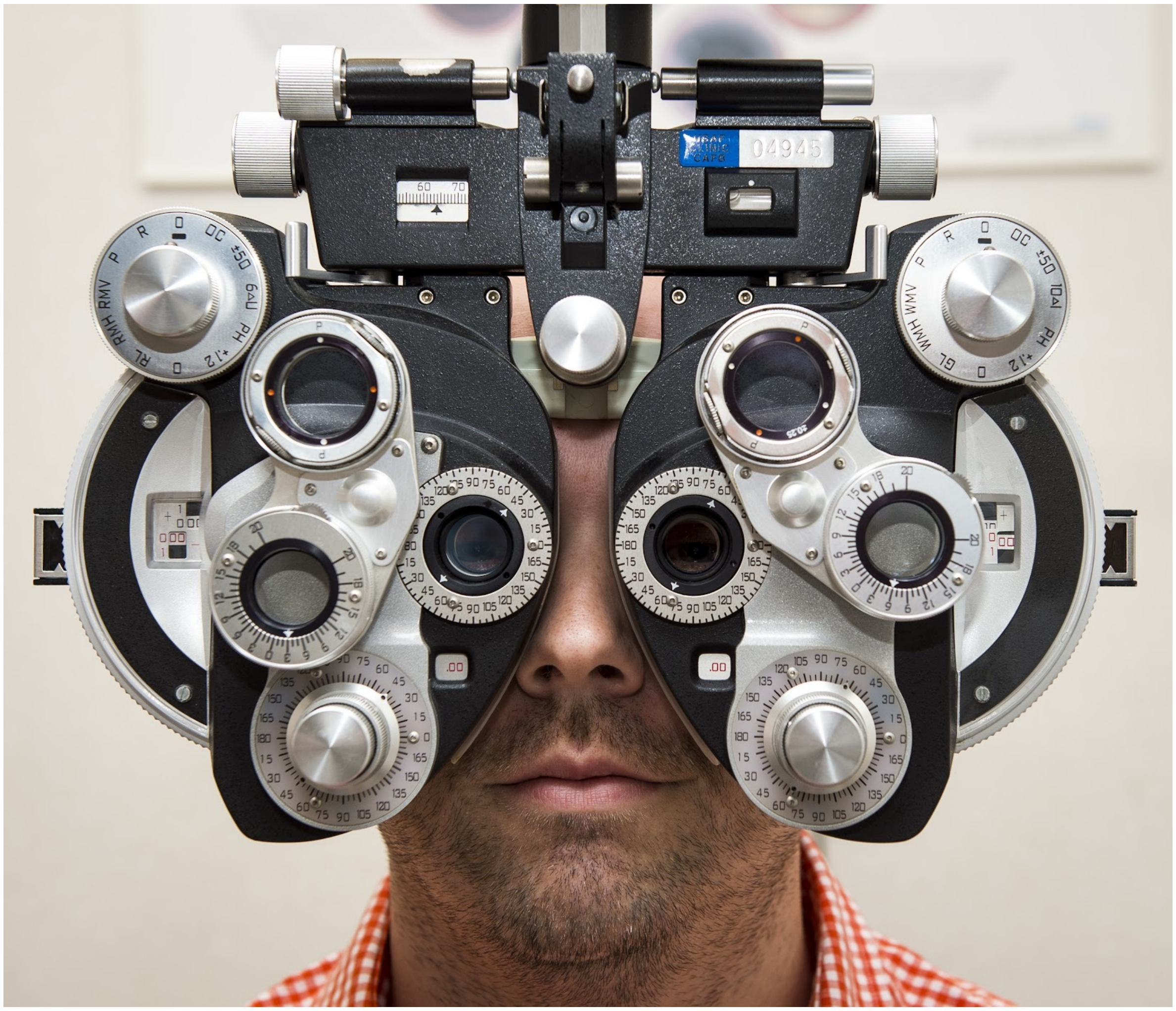

* Continue

◯ Yes

◯ No

Eye Research Priority Survey

Optometry Research Priorities

In this section, we are asking you to indicate which research priority questions you feel are important within Optometry Research. You will then be asked to rank each research question selected in order of priority.

* **Please select each research question you feel is a high priority**

- [ ] **Does the enhanced integration of ophthalmic primary and secondary care via community optometric care pathways lead to improved detection, treatment and management of eye disease, and to improved patient quality of life?** - *In the UK individuals with eye diseases traditionally receive care in hospital eye clinics (secondary care). These NHS services are however under great strain and many patients may benefit from management by optometrists in their community (in primary care). While some initial research has demonstrated the benefit of these schemes, further work is needed to determine if such novel eye-care pathways will benefit patient care and the public more widely by reducing costs and waiting times, while also improving patient satisfaction and quality of life.*

- [ ] **How can the detection, diagnosis and management of ocular surface disorders be improved?** - *Ocular surface disorders include a range of conditions where the outermost layer of the front of the eye becomes damaged. This can be caused by problems in the eye or disease elsewhere in the body and may lead to reductions in vision and quality of life. New methods for the diagnosis, monitoring and treatment of ocular surface disorders are required.*

- [ ] **Can corneal infections be prevented in high-risk individuals such as contact lens wearers?** - *The cornea is the clear, dome-shaped tissue on the front of the eye. Infection and damage of this tissue can lead to profound and often permanent reductions in vision, in addition to marked pain and discomfort. Further research is needed to identify why such conditions develop and how they may be prevented.*

☐ **How can novel medical devices and technology be applied to improve the prevention, diagnosis, and management of eye disease?** - *Many new clinical instruments and specialist computer programmes that interpret clinical data (e.g., Artificial Intelligence systems) are being developed. While promising, research is required to understand how these tools may be best applied for the prevention, diagnosis, and management of eye diseases, to ensure that they are safe, and to determine if they will improve eye-care services.*

☐ **What are the most appropriate measures of visual function, structure and vision-related quality-of-life for the detection and monitoring of cataracts?** - Cataract is one of the most common causes of reduced vision worldwide. It occurs when the lens of the eye becomes cloudy. This condition can be managed effectively with surgery to remove the cloudy lens, which improves both vision and quality of life. However, it is unclear what clinical tests are most sensitive to reliably detect the presence of cataracts and help understand the effects of this on both vision and quality of life. As a result, further research is needed to identify the best clinical tests for cataract assessment to ensure patients with this condition are offered surgical treatment at the right time.

☐ **What is the most effective management of ocular complications associated with Stevens Johnson Syndrome?** - *Stevens Johnson Syndrome is a rare but very serious disorder that leads to damage to the soft layer of tissues that lines the eye and other areas of the body (mucous membranes). This can lead to problems in the cornea (clear, dome-shaped tissue on the front of the eye) such as ulcers and scarring, with resulting vision loss. There is a need to identify methods to effectively manage such complications to improve patient vision and comfort.*

Eye Research Priority Survey

Optometry Research Priorities

In this section, we are asking you to indicate which research priority questions you feel are important within Optometry Research. You will then be asked to rank each research question selected in order of priority.

*** Please could you now rank the high priority questions selected in the previous question in order of priority? The questions can be dragged and dropped or ranked with one being the highest and six being the lowest.**

Does the enhanced integration of ophthalmic primary and secondary care via community optometric care pathways lead to improved detection, treatment and management of eye disease, and to improved patient quality of life? - *In the UK individuals with eye diseases traditionally receive care in hospital eye clinics (secondary care). These NHS services are however under great strain and many patients may benefit from management by optometrists in their community (in primary care). While some initial research has demonstrated the benefit of these schemes, further work is needed to determine if such novel eye-care pathways will benefit patient care and the public more widely by reducing costs and waiting times, while also improving patient satisfaction and quality of life.*

How can novel medical devices and technology be applied to improve the prevention, diagnosis, and management of eye disease?- *Many new clinical instruments and specialist computer programmes that interpret clinical data (e.g., Artificial Intelligence systems) are being developed. While promising, research is required to understand how these tools may be best applied for the prevention, diagnosis, and management of eye diseases, to ensure that they are safe, and to determine if they will improve eye-care services.*

What are the most appropriate measures of visual function, structure and vision-related quality-of-life for the detection and monitoring of cataracts? - Cataract is one of the most common causes of reduced vision worldwide. It occurs when the lens of the eye becomes cloudy. This condition can be managed effectively with surgery to remove the cloudy lens, which improves both vision and quality of life. However, it is unclear what clinical tests are most sensitive to reliably detect the presence of cataracts and help understand the effects of this on both vision and quality of life. As a result, further research is needed to identify the best clinical tests for cataract assessment to ensure patients with this condition are offered surgical treatment at the right time.

How can the detection, diagnosis and management of ocular surface disorders be improved? - *Ocular surface disorders include a range of conditions where the outermost layer of the front of the eye becomes damaged. This can be caused by problems in the eye or disease elsewhere in the body and may lead to reductions in vision and quality of life. New methods for the diagnosis, monitoring and treatment of ocular surface disorders are required.*

Can corneal infections be prevented in high-risk individuals such as contact lens wearers? - *The cornea is the clear, dome-shaped tissue on the front of the eye. Infection and damage of this tissue can lead to profound and often permanent reductions in vision, in addition to marked pain and discomfort. Further research is needed to identify why such conditions develop and how they may be prevented.*

What is the most effective management of ocular complications associated with Stevens Johnson Syndrome? - *Stevens Johnson Syndrome is a rare but very serious disorder that leads to damage to the soft layer of tissues that lines the eye and other areas of the body (mucous membranes). This can lead to problems in the cornea (clear, dome-shaped tissue on the front of the eye) such as ulcers and scarring, with resulting vision loss. There is a need to identify methods to effectively manage such complications to improve patient vision and comfort.*

Eye Research Priority Survey

Cornea Research: What should the research priorities be?

An eye research priority setting exercise being undertaken through the UK Clinical Eye Research Strategy. The aim of this priority setting exercise is to update the hugely useful James Lind Alliance (JLA) Priority Setting Partnership (PSP) for Sight Loss and Vision exercise of 2013 and help guide eye research in the future.

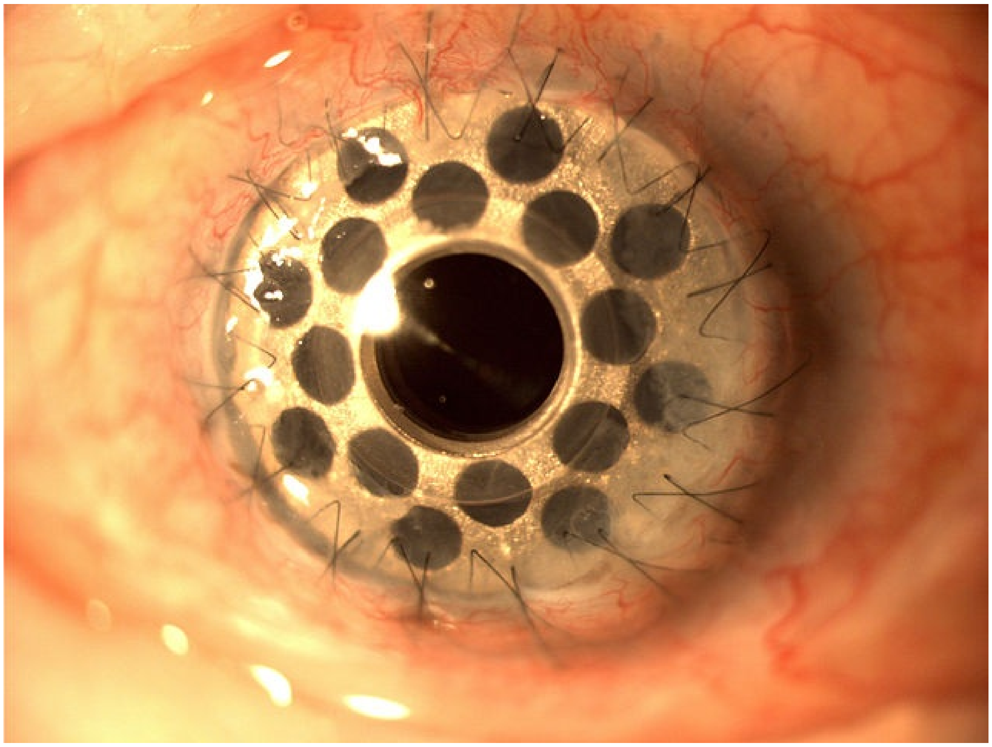

*** Continue**

◯ Yes

◯ No

Eye Research Priority Survey

Cornea Research Priorities

In this section, we are asking you to indicate which research priority questions you feel are important within Cornea Research. You will then be asked to rank each research question selected in order of priority.

*** Please select each research question you feel is a high priority up to a maximum of ten questions.**

- [ ] **How to standardize the diagnosis and monitoring of dry eye?** - *Dry eye disease is a common condition that occurs when your tears aren't able to provide adequate lubrication for your eyes.*

- [ ] **How can dry eye treatment be improved?** - *Dry eye disease is a common condition that occurs when your tears aren't able to provide adequate lubrication for your eyes.*

- [ ] **How can microbial keratitis treatment be improved?** - *Microbial keratitis is an infection of the cornea (the clear dome covering the coloured part of the eye) that is caused by microbe. It can affect contact lens wearers, and also sometimes people who do not wear contact lenses.*

- [ ] **How can diagnosis of corneal infections be improved and how can corneal infection be prevented in high-risk individuals?** - *Microbial keratitis is an infection of the cornea (the clear dome covering the coloured part of the eye) that is caused by microbe. It can affect contact lens wearers, and also sometimes people who do not wear contact lenses.*

- [ ] **How can detection of progression in Keratoconus patients be improved** - *Keratoconus is an eye disease that affects the structure of the cornea, resulting in loss of vision. It occurs when your cornea — the clear, dome-shaped front surface of your eye — thins and gradually bulges outward into a cone shape.*

- [ ] **How can we prevent Keratoconus progression?** - *Keratoconus is an eye disease that affects the structure of the cornea, resulting in loss of vision. It occurs when your cornea — the clear, dome-shaped front surface of your eye — thins and gradually bulges outward into a cone shape.*

- [ ] **How can quality of life of contact lenses wearer for Keratoconus disease be improved ?** - *Keratoconus is an eye disease that affects the structure of the cornea, resulting in loss of vision. It occurs when your cornea — the clear, dome-shaped front surface of your eye — thins and gradually bulges outward into a cone shape.*

- [ ] **How can the rejection of corneal transplants be prevented?** - *Rejection happens when your immune system recognises the donated cornea as not belonging to you and attacks it.*

- [ ] **How can corneal transplant complication related to vaccinations be improved ?** - *A cornea transplant is an operation to remove all or part of a damaged cornea and replace it with healthy donor tissue. It can be used to improve sight, relieve pain and treat severe infection or damage. Vaccinations seems to increase the postoperative complication.*

- [ ] **How can visual outcomes of corneal transplantation be improved?** - *A cornea transplant is an operation to remove all or part of a damaged cornea and replace it with healthy donor tissue.*

- [ ] **How can utilization of corneal donor tissues be improved?** - *Cornea donation is necessary for the preservation and restoration of sight. One cornea donor can restore the sight of two people*

- [ ] **What is the cause of Keratoconus and can it be prevented?** - *Keratoconus is an eye disease that affects the structure of the cornea, resulting in loss of vision.*

- [ ] **How can non-surgical therapy for Corneal endothelial dysfunctions be developed?** - *Corneal endothelial dysfunction occurs when corneal endothelial cells (CECs) are dramatically lost and eventually results in vision loss. Corneal transplantation is the only solution at present. However, corneal transplantation requires a fresh human cornea and there is a worldwide shortage of donors.*

- [ ] **How can ocular complications associated with Stevens Johnson Syndrome be improved ?** - *Stevens-Johnson syndrome (SJS) is a rare, serious disorder of the skin and mucous membranes. It's usually a reaction to medication that starts with flu-like symptoms, followed by a painful rash that spreads and blisters.*

- [ ] **How can ocular surface disease in children, such as blepharokeratoconjunctivitis and vernal keratoconjunctivitis be managed better?** - *Blepharokeratoconjunctivitis is a chronic inflammatory disease of the palpebral margin with secondary conjunctival and corneal involvement that affects the paediatric population. Vernal keratoconjunctivitis is an allergic eye disease that especially affects young boys. The most common symptoms are itching, photophobia, burning, and tearing.*

- [ ] **How can telemedicine be improved for diagnosis, management and treatment of ocular surface disease ?** - *Ocular surface diseases are disease that affected the surface layers of the eye*

Eye Research Priority Survey

Cornea Research Priorities

In this section, we are asking you to indicate which research priority questions you feel are important within Cornea Research. You will then be asked to rank each research question selected in order of priority.

*** Please could you now rank the high priority questions selected in the previous question in order of priority? The questions can be dragged and dropped or ranked with one being the highest and ten being the lowest.**

- **How to standardize the diagnosis and monitoring of dry eye?** - *Dry eye disease is a common condition that occurs when your tears aren't able to provide adequate lubrication for your eyes.*
- **How can dry eye treatment be improved?** - *Dry eye disease is a common condition that occurs when your tears aren't able to provide adequate lubrication for your eyes.*
- **How can microbial keratitis treatment be improved?** - *Microbial keratitis is an infection of the cornea (the clear dome covering the coloured part of the eye) that is caused by microbe. It can affect contact lens wearers, and also sometimes people who do not wear contact lenses.*
- **How can diagnosis of corneal infections be improved and how can corneal infection be prevented in high-risk individuals?** - *Microbial keratitis is an infection of the cornea (the clear dome covering the coloured part of the eye) that is caused by microbe. It can affect contact lens wearers, and also sometimes people who do not wear contact lenses.*
- **How can the rejection of corneal transplants be prevented?** - *Rejection happens when your immune system recognises the donated cornea as not belonging to you and attacks it.*
- **How can corneal transplant complication related to vaccinations be improved ?** - *A cornea transplant is an operation to remove all or part of a damaged cornea and replace it with healthy donor tissue. It can be used to improve sight, relieve pain and treat severe infection or damage. Vaccinations seems to increase the postoperative complication.*
- **How can visual outcomes of corneal transplantation be improved?** - *A cornea transplant is an operation to remove all or part of a damaged cornea and replace it with healthy donor tissue.*

≣ **[How can utilization of corneal donor tissues be improved?]** - *Cornea donation is necessary for the preservation and restoration of sight. One cornea donor can restore the sight of two people*

≣ **[How can detection of progression in Keratoconus patients be improved]** - *Keratoconus is an eye disease that affects the structure of the cornea, resulting in loss of vision. It occurs when your cornea — the clear, dome-shaped front surface of your eye — thins and gradually bulges outward into a cone shape.*

≣ **[How can we prevent Keratoconus progression?]** - *Keratoconus is an eye disease that affects the structure of the cornea, resulting in loss of vision. It occurs when your cornea — the clear, dome-shaped front surface of your eye — thins and gradually bulges outward into a cone shape.*

≣ **[How can quality of life of contact lenses wearer for Keratoconus disease be improved ?]** - *Keratoconus is an eye disease that affects the structure of the cornea, resulting in loss of vision. It occurs when your cornea — the clear, dome-shaped front surface of your eye — thins and gradually bulges outward into a cone shape.*

≣ **[What is the cause of Keratoconus and can it be prevented?]** - *Keratoconus is an eye disease that affects the structure of the cornea, resulting in loss of vision.*

≣ **[How can non-surgical therapy for Corneal endothelial dysfunctions be developed?]** - *Corneal endothelial dysfunction occurs when corneal endothelial cells (CECs) are dramatically lost and eventually results in vision loss. Corneal transplantation is the only solution at present. However, corneal transplantation requires a fresh human cornea and there is a worldwide shortage of donors.*

≣ **[How can ocular complications associated with Stevens Johnson Syndrome be improved ?]** - *Stevens-Johnson syndrome (SJS) is a rare, serious disorder of the skin and mucous membranes. It's usually a reaction to medication that starts with flu-like symptoms, followed by a painful rash that spreads and blisters.*

≣ **[How can ocular surface disease in children, such as blepharokeratoconjunctivitis and vernal keratoconjunctivitis be managed better?]** - *Blepharokeratoconjunctivitis is a chronic inflammatory disease of the palpebral margin with secondary conjunctival and corneal involvement that affects the paediatric population. Vernal keratoconjunctivitis is an allergic eye disease that especially affects young boys. The most common symptoms are itching, photophobia, burning, and tearing.*

≣ **[How can telemedicine be improved for diagnosis, management and treatment of ocular surface disease ?]** - *Ocular surface diseases are disease that affected the surface layers of the eye*

Eye Research Priority Survey

Cataract Research: What should the research priorities be?

An eye research priority setting exercise being undertaken through the UK Clinical Eye Research Strategy. The aim of this priority setting exercise is to update the hugely useful James Lind Alliance (JLA) Priority Setting Partnership (PSP) for Sight Loss and Vision exercise of 2013 and help guide eye research in the future.

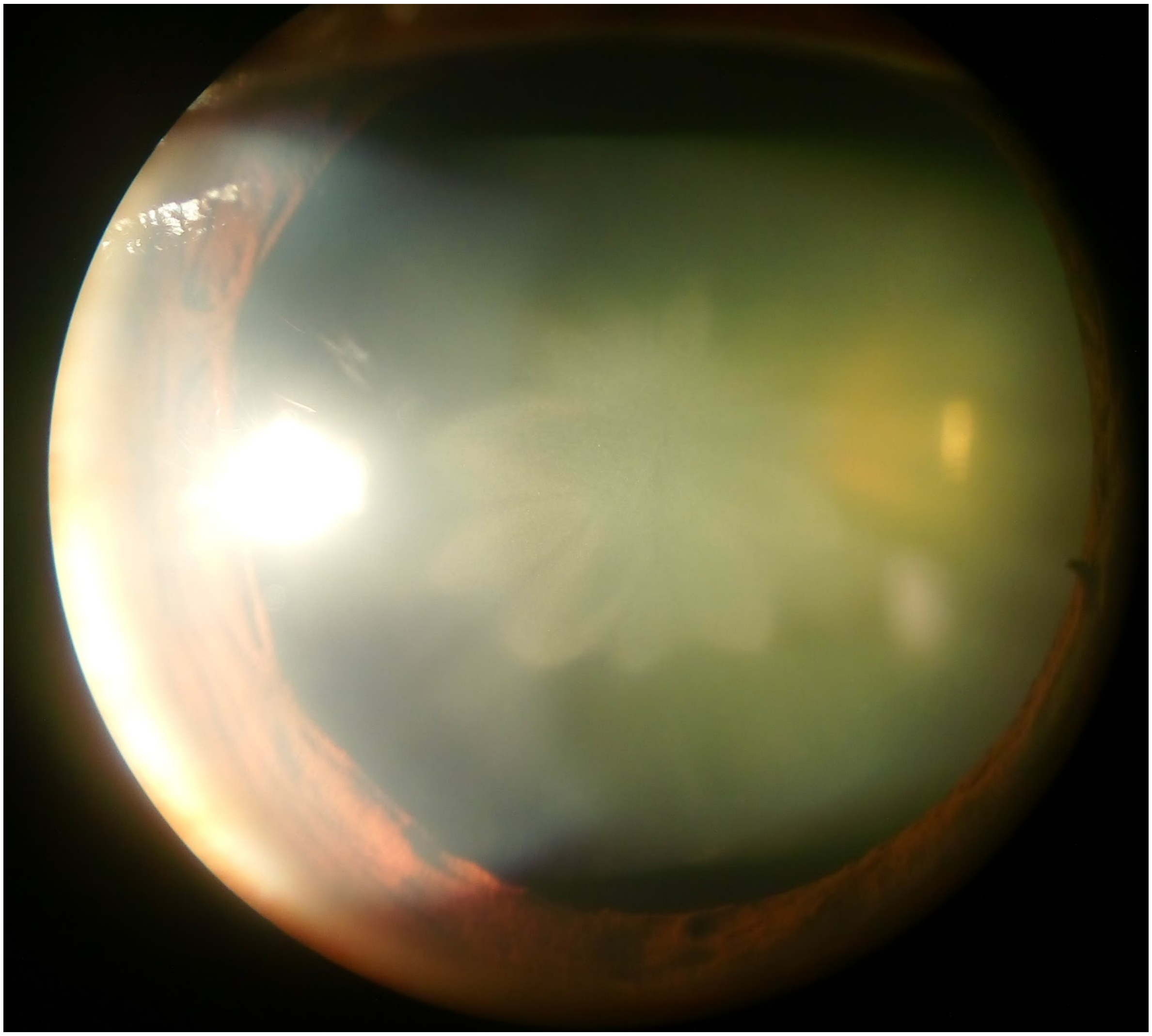

* Continue

◯ Yes

◯ No

Eye Research Priority Survey

Cataract Research Priorities

In this section, we are asking you to indicate which research priority questions you feel are important within Cataract Research. You will then be asked to rank each research question selected in order of priority.

* Please select each research question you feel is a high priority up to a maximum of ten questions.

- [ ] **What is the cause of cataract? How do cataracts form?** - *What is the effect of sunlight on the development of cataract? What is the effect of excess alcohol intake on cataract formation?*

- [ ] **How can cataracts be prevented from developing?** - *What can be done in earlier life to prevent cataracts developing? Are there any lifestyle changes or dietary measures that can be taken to prevent cataract?*

- [ ] **How can cataract progression be slowed down?** - *Can dietary measures, nutritional supplements or complementary therapies slow down the progression of cataracts?*

- [ ] **What alternatives to treat cataracts other than cataract surgery are being developed?**

- [ ] **How can cataract surgery outcomes be improved?** - *Are there any ways, using technology, systems, processes, primary care or other, which can help improve the surgery, process of surgery and results?*

- [ ] **What are the roles of telemedicine/remote medicine, AI, electronic patient records, smart theatres, OCT, biometry and other technologies in the future of cataract care?** - *Should we be looking at developing or using certain emerging or existing technologies in cataract care*

- [ ] **Should we be looking at developing or using certain emerging or existing technologies in cataract care** - *The pandemic has brought about many changes, some of which may benefit patients – should we be looking to offer these new services in the long term?*

- [ ] **How safe is bilateral simultaneous surgery?** - *The majority of cataract surgery is done one eye at a time. There are a number of reasons for this. Should we explore doing both eyes at the same sitting, and how safe is this?*

- [ ] **Should accommodative lenses be developed for cataract surgery?** - *The implanted lenses used during cataract surgery do not exactly replicate the vision we have in youth. They do not have a range of focus, like people do prior to midlife. Should we be looking to develop such lenses?*

- [ ] **What is the best measure of visual disability due to cataract?** - *What is the most effective way to monitor development of cataract?*

- [ ] **Can the return of cloudy or blurred vision after cataract surgery known as posterior capsule opacity (PCO) or secondary cataract be prevented?** - *One of the commonest consequences of cataract surgery is to develop cloudiness in the natural biological sack that holds the implant in the eye (known as PCO). This requires laser removal. Should we continue to look at ways of preventing this problem?*

- [ ] **Can retinal detachment be prevented after cataract surgery?** - *Retinal detachment is a serious condition, which is known to happen more frequently post cataract surgery. Should we be looking at ways to prevent this?*

- [ ] **What are the outcomes for cataract surgery among people with different levels of cognitive impairment (whatever the cause but including dementia, stroke, neurological conditions, head injuries)?** - *Should we be specifically evaluating the results of cataract surgery in different groups of patients who either have specific needs or in whom some of the standard tests may not give a fully representative picture?*

Eye Research Priority Survey

Cataract Research Priorities

In this section, we are asking you to indicate which research priority questions you feel are important within Cataract Research. You will then be asked to rank each research question selected in order of priority.

*** Please could you now rank the high priority questions selected in the previous question in order of priority? The questions can be dragged and dropped or ranked with one being the highest and ten being the lowest.**

- **What is the cause of cataract? How do cataracts form?** - *What is the effect of sunlight on the development of cataract? What is the effect of excess alcohol intake on cataract formation?*
- **How can cataracts be prevented from developing?** - *What can be done in earlier life to prevent cataracts developing? Are there any lifestyle changes or dietary measures that can be taken to prevent cataract?*
- **How can cataract progression be slowed down?** - *Can dietary measures, nutritional supplements or complementary therapies slow down the progression of cataracts?*
- **What alternatives to treat cataracts other than cataract surgery are being developed?**
- **How can cataract surgery outcomes be improved?** - *Are there any ways, using technology, systems, processes, primary care or other, which can help improve the surgery, process of surgery and results?*
- **What are the roles of telemedicine/remote medicine, AI, electronic patient records, smart theatres, OCT, biometry and other technologies in the future of cataract care?** - *Should we be looking at developing or using certain emerging or existing technologies in cataract care*
- **Should we be looking at developing or using certain emerging or existing technologies in cataract care** - *The pandemic has brought about many changes, some of which may benefit patients – should we be looking to offer these new services in the long term?*
- **How safe is bilateral simultaneous surgery?** - *The majority of cataract surgery is done one eye at a time. There are a number of reasons for this. Should we explore doing both eyes at the same sitting, and how safe is this?*
- **Should accommodative lenses be developed for cataract surgery?** - *The implanted lenses used during cataract surgery do not exactly replicate the vision we have in youth. They do not have a range of focus, like people do prior to midlife. Should we be looking to develop such lenses?*
- **What is the best measure of visual disability due to cataract?** - *What is the most effective way to monitor development of cataract?*
- **Can the return of cloudy or blurred vision after cataract surgery known as posterior capsule opacity (PCO) or secondary cataract be prevented?** - *One of the commonest consequences of cataract surgery is to develop cloudiness in the natural biological sack that holds the implant in the eye (known as PCO). This requires laser removal. Should we continue to look at ways of preventing this problem?*
- **Can retinal detachment be prevented after cataract surgery?** - *Retinal detachment is a serious condition, which is known to happen more frequently post cataract surgery. Should we be looking at ways to prevent this?*

What are the outcomes for cataract surgery among people with different levels of cognitive impairment (whatever the cause but including dementia, stroke, neurological conditions, head injuries)? - *Should we be specifically evaluating the results of cataract surgery in different groups of patients who either have specific needs or in whom some of the standard tests may not give a fully representative picture?*

Eye Research Priority Survey

Glaucoma Research: What should the research priorities be?

An eye research priority setting exercise being undertaken through the UK Clinical Eye Research Strategy. The aim of this priority setting exercise is to update the hugely useful James Lind Alliance (JLA) Priority Setting Partnership (PSP) for Sight Loss and Vision exercise of 2013 and help guide eye research in the future.

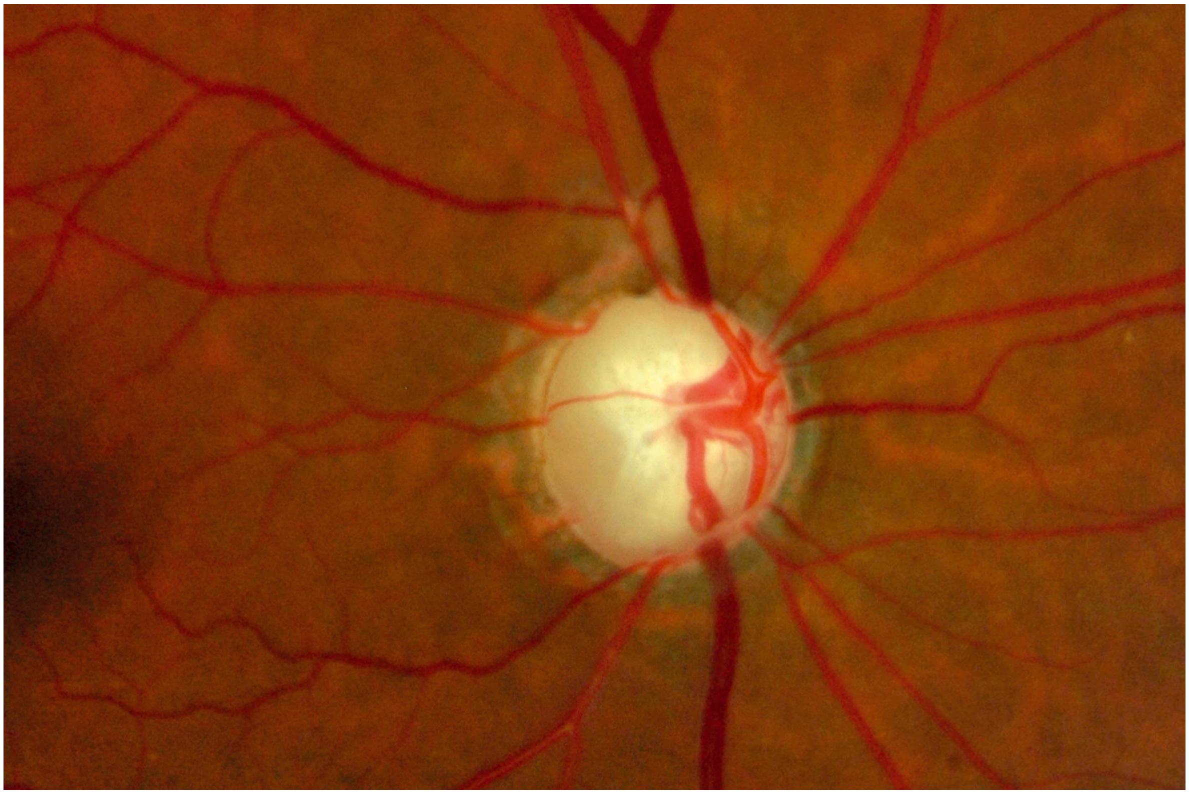

*** Continue**

◯ Yes

◯ No

Eye Research Priority Survey

Glaucoma Research Priorities

In this section, we are asking you to indicate which research priority questions you feel are important within Glaucoma Research. You will then be asked to rank each research question selected in order of priority.

* **Please select each research question you feel is a high priority**

- [ ] **What are the most effective treatments for glaucoma and how can treatment be improved?** - *There are different options to treat people with glaucoma, including eye drops, laser, and surgery. Treatments are individualised according to risk of visual loss and patients' circumstances, including personal preferences. Recent developments in medical and surgical treatments for glaucoma have increased the number of possible options. There is a need to compare the effectiveness of various treatments and determine at what stage of disease and in which patients they work best.*

- [ ] **How can any vision loss be restored for people with glaucoma?** - *Although treatment for glaucoma is often effective to halt or slow disease progression, at the moment we don't have treatments that can improve vision in people with glaucomatous visual loss. Experimental studies have found that the use of stem cells or strategies for repairing or regenerating retinal cells damaged in glaucoma can potentially lead to new treatments.*

- [ ] **What causes glaucoma?** - *Better understanding of the mechanisms responsible for the different types of glaucoma will be helpful to identify novel ways of preventing or treating the disease. Of particular interest would be, for example, improved knowledge of the reasons why glaucoma is more common and more severe in some ethnic groups, such as those of West African origin, or the role of genetic factors, or the associations between glaucoma and blood pressure.*

- [ ] **What is the most effective way of monitoring the progression of glaucoma?** - *Glaucoma is a chronic disease, and people with glaucoma need life-long monitoring to detect when and how quickly the disease is progressing. We would like to have better evidence on the type of tests, frequency of testing, setting (hospital vs community vs home) and optimal strategies to monitor disease progression.*

- [ ] **What can be done to avoid late diagnosis of sight-threatening glaucoma?** - *Severity of glaucoma at diagnosis is one of the most important risk factors for severe visual loss. In the UK most people with glaucoma are unaware that they have it. There is substantial room to improve current strategies to detect glaucoma. Diagnosis of glaucoma at an early stage is important so treatment can be started and visual loss prevented. Detecting glaucoma may be difficult due to the lack of symptoms in early stages, a failure to access eyecare at the right time, and also because the diagnostic tests for glaucoma are imperfect. Overall affects 2% of the adult population above 40 years of age. It is more common in elderly people, among those with a relative with glaucoma, and in some ethnic groups (e.g., those of West African origin). We don't know how best to improve the detection of people with glaucoma in the community, e.g., - how can we encourage people to have regular eye exams, how primary care should be organised, e.g., at what age should people be tested, what test should be used, should newer devices such as optical coherence tomography be used for glaucoma detection, what is the role of genetic testing or artificial intelligence for identifying those at greater risk of glaucoma.*

- [ ] **Is there a link between treatment adherence and glaucoma progression and how can adherence be improved?** - *The most common treatment for glaucoma is topical eye drops. Due to the long-term need for treatment, it is important that people can adhere to the recommended regime. However, many people have problems using eye drops and we don't have good evidence to know how to evaluate and improve adherence.*

Eye Research Priority Survey

Glaucoma Research Priorities

In this section, we are asking you to indicate which research priority questions you feel are important within Glaucoma Research. You will then be asked to rank each research question selected in order of priority.

*** Please could you now rank the high priority questions selected in the previous question in order of priority? The questions can be dragged and dropped or ranked with one being the highest and six being the lowest.**

- **What are the most effective treatments for glaucoma and how can treatment be improved?** - *There are different options to treat people with glaucoma, including eye drops, laser, and surgery. Treatments are individualised according to risk of visual loss and patients' circumstances, including personal preferences. Recent developments in medical and surgical treatments for glaucoma have increased the number of possible options. There is a need to compare the effectiveness of various treatments and determine at what stage of disease and in which patients they work best.*
- **How can any vision loss be restored for people with glaucoma?** - *Although treatment for glaucoma is often effective to halt or slow disease progression, at the moment we don't have treatments that can improve vision in people with glaucomatous visual loss. Experimental studies have found that the use of stem cells or strategies for repairing or regenerating retinal cells damaged in glaucoma can potentially lead to new treatments.*
- **What can be done to avoid late diagnosis of sight-threatening glaucoma?** - *Severity of glaucoma at diagnosis is one of the most important risk factors for severe visual loss. In the UK most people with glaucoma are unaware that they have it. There is substantial room to improve current strategies to detect glaucoma. Diagnosis of glaucoma at an early stage is important so treatment can be started and visual loss prevented. Detecting glaucoma may be difficult due to the lack of symptoms in early stages, a failure to access eyecare at the right time, and also because the diagnostic tests for glaucoma are imperfect. Overall affects 2% of the adult population above 40 years of age. It is more common in elderly people, among those with a relative with glaucoma, and in some ethnic groups (e.g., those of West African origin). We don't know how best to improve the detection of people with glaucoma in the community, e.g., - how can we encourage people to have regular eye exams, how primary care should be organised, e.g., at what age should people be tested, what test should be used, should newer devices such as optical coherence tomography be used for glaucoma detection, what is the role of genetic testing or artificial intelligence for identifying those at greater risk of glaucoma.*
- **What causes glaucoma?** - *Better understanding of the mechanisms responsible for the different types of glaucoma will be helpful to identify novel ways of preventing or treating the disease. Of particular interest would be, for example, improved knowledge of the reasons why glaucoma is more common and more severe in some ethnic groups, such as those of West African origin, or the role of genetic factors, or the associations between glaucoma and blood pressure.*
- **What is the most effective way of monitoring the progression of glaucoma?** - *Glaucoma is a chronic disease, and people with glaucoma need life-long monitoring to detect when and how quickly the disease is progressing. We would like to have better evidence on the type of tests, frequency of testing, setting (hospital vs community vs home) and optimal strategies to monitor disease progression.*
- **Is there a link between treatment adherence and glaucoma progression and how can adherence be improved?** - *The most common treatment for glaucoma is topical eye drops. Due to the long-term need for treatment, it is important that people can adhere to the recommended regime. However, many people have problems using eye drops and we don't have good evidence to know how to evaluate and improve adherence.*

Eye Research Priority Survey

Retinal Research: What should the research priorities be?

An eye research priority setting exercise being undertaken through the UK Clinical Eye Research Strategy. The aim of this priority setting exercise is to update the hugely useful James Lind Alliance (JLA) Priority Setting Partnership (PSP) for Sight Loss and Vision exercise of 2013 and help guide eye research in the future.

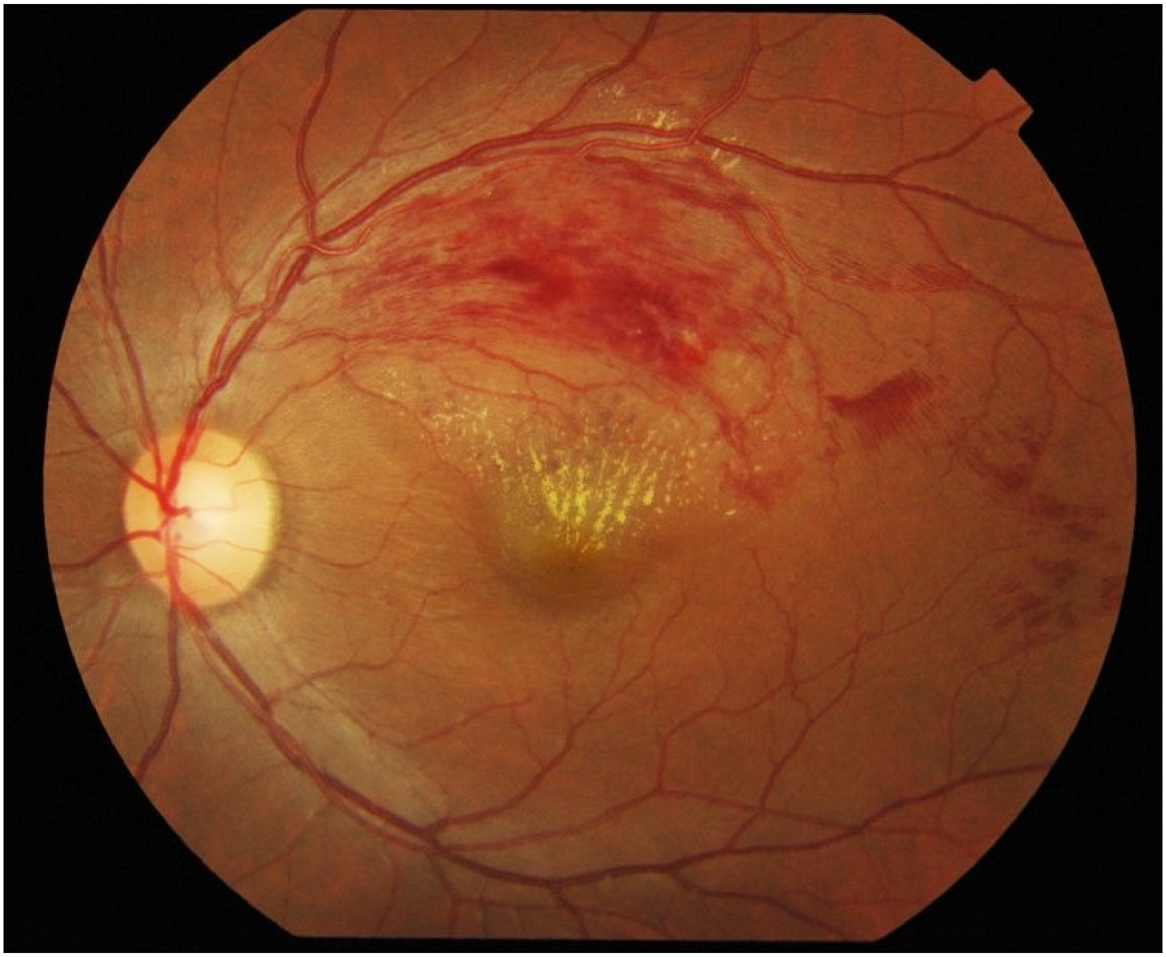

* **Continue**

◯ Yes

◯ No

Eye Research Priority Survey

Retinal Research Priorities

In this section, we are asking you to indicate which research priority questions you feel are important within Retinal Research. You will then be asked to rank each research question selected in order of priority.

*** Please select each research question you feel is a high priority up to a maximum of ten questions.**

- [ ] **How can the prevention, diagnosis and treatment of wet age related macular degeneration be improved?** - *Wet age related macular degeneration is a common cause of central vision loss in the elderly. Although treatment is available for this condition, the burden of treatment, costs and unsustainable treatment effects call for better management of this condition.*

- [ ] **How can the prevention, diagnosis and treatment of dry age related macular degeneration be improved?** - *Advanced dry age related macular degeneration is the commonest cause of central vision loss in the elderly. There are no treatments for this condition.*

- [ ] **How can the prevention, diagnosis and treatment of diabetic eye disease be improved?** - *Retinal blood vessels become unhealthy due to diabetes and they can leak or die off. New treatments are required to prevent the blood vessels from becoming unhealthy or to treat them before they develop complications.*

- [ ] **How can the prevention, diagnosis and treatment of macular holes be improved?** - *Macular holes cause central vision loss. There are unanswered questions:What is the most effective and safe treatment for macular holes? Are there environmental or genetic factors that predispose to macular holes?*

- [ ] **How can the prevention, diagnosis and treatment of ocular melanoma be improved?** - *Cancer can occur in the eye first or affect the eye from other parts of the body.*

- [ ] **How can sight loss due to inherited retinal diseases be prevented or restored?** - *Specific unanswered questions include: Can genetic screening be done for all these conditions and diagnosis and prognosis provided early? Can stem cell and gene therapy help all inherited retinal diseases?*

- [ ] **How can the prevention, diagnosis and treatment of ocular inflammatory disease be improved?** - *Uveitis is inflammation of the vascular bed of the eye and it can get inflamed primarily in the eye or from diseases from other parts of the body. Steroids are the mainstay for this condition but it is associated with side-effects.*

- [ ] **Visual rehabilitation in eyes with central visual loss due to retinal diseases?** - *Several new electronic and other devices are now available for visual rehabilitation. It is unclear how effective they are and so a comparative study of the various gadgets is required.*

- [ ] **Artificial Intelligence in retinal diseases** - *Automated diagnosis and prognosis are now possible with artificial intelligence algorithms. Before implementation, the clinical and cost-effectiveness of these algorithms as decision tools needs to be compared to standard care.*

☐ **[How can the prevention, diagnosis and treatment of fibrosis as a complication of retinal diseases be improved?]** - *Fibrosis is an adverse reaction of the eye to an injury or disease and currently, there are several questions that need to be answered: How can surgical techniques be improved to save sight for eyes damaged by injury?, How can the risk of losing sight for people with retinal detachment be reduced? How can the success rate of surgery for retinal detachment be improved? How can epiretinal membrane/fibrosis be prevented or treated?*

Eye Research Priority Survey

Retinal Research Priorities

In this section, we are asking you to indicate which research priority questions you feel are important within Retinal Research. You will then be asked to rank each research question selected in order of priority.

*** Please could you now rank the high priority questions selected in the previous question in order of priority? The questions can be dragged and dropped or ranked with one being the highest and ten being the lowest.**

- **How can the prevention, diagnosis and treatment of wet age related macular degeneration be improved?** - *Wet age related macular degeneration is a common cause of central vision loss in the elderly. Although treatment is available for this condition, the burden of treatment, costs and unsustainable treatment effects call for better management of this condition.*
- **How can the prevention, diagnosis and treatment of dry age related macular degeneration be improved?** - *Advanced dry age related macular degeneration is the commonest cause of central vision loss in the elderly. There are no treatments for this condition.*
- **How can the prevention, diagnosis and treatment of diabetic eye disease be improved?** - *Retinal blood vessels become unhealthy due to diabetes and they can leak or die off. New treatments are required to prevent the blood vessels from becoming unhealthy or to treat them before they develop complications.*
- **How can the prevention, diagnosis and treatment of macular holes be improved?** - *Macular holes cause central vision loss. There are unanswered questions:What is the most effective and safe treatment for macular holes? Are there environmental or genetic factors that predispose to macular holes?*
- **How can the prevention, diagnosis and treatment of fibrosis as a complication of retinal diseases be improved?** - *Fibrosis is an adverse reaction of the eye to an injury or disease and currently, there are several questions that need to be answered: How can surgical techniques be improved to save sight for eyes damaged by injury?, How can the risk of losing sight for people with retinal detachment be reduced? How can the success rate of surgery for retinal detachment be improved? How can epiretinal membrane/fibrosis be prevented or treated?*
- **How can the prevention, diagnosis and treatment of ocular melanoma be improved?** - *Cancer can occur in the eye first or affect the eye from other parts of the body.*
- **How can sight loss due to inherited retinal diseases be prevented or restored?** - *Specific unanswered questions include: Can genetic screening be done for all these conditions and diagnosis and prognosis provided early? Can stem cell and gene therapy help all inherited retinal diseases?*
- **How can the prevention, diagnosis and treatment of ocular inflammatory disease be improved?** - *Uveitis is inflammation of the vascular bed of the eye and it can get inflamed primarily in the eye or from diseases from other parts of the body. Steroids are the mainstay for this condition but it is associated with side-effects.*
- **Visual rehabilitation in eyes with central visual loss due to retinal diseases?** - *Several new electronic and other devices are now available for visual rehabilitation. It is unclear how effective they are and so a comparative study of the various gadgets is required.*
- **Artificial Intelligence in retinal diseases** - *Automated diagnosis and prognosis are now possible with artificial intelligence algorithms. Before implementation, the clinical and cost-effectiveness of these algorithms as decision tools needs to be compared to standard care.*

Eye Research Priority Survey

Childhood-Onset Eye Disorders Research: What should the research priorities be?

An eye research priority setting exercise being undertaken through the UK Clinical Eye Research Strategy. The aim of this priority setting exercise is to update the hugely useful James Lind Alliance (JLA) Priority Setting Partnership (PSP) for Sight Loss and Vision exercise of 2013 and help guide eye research in the future.

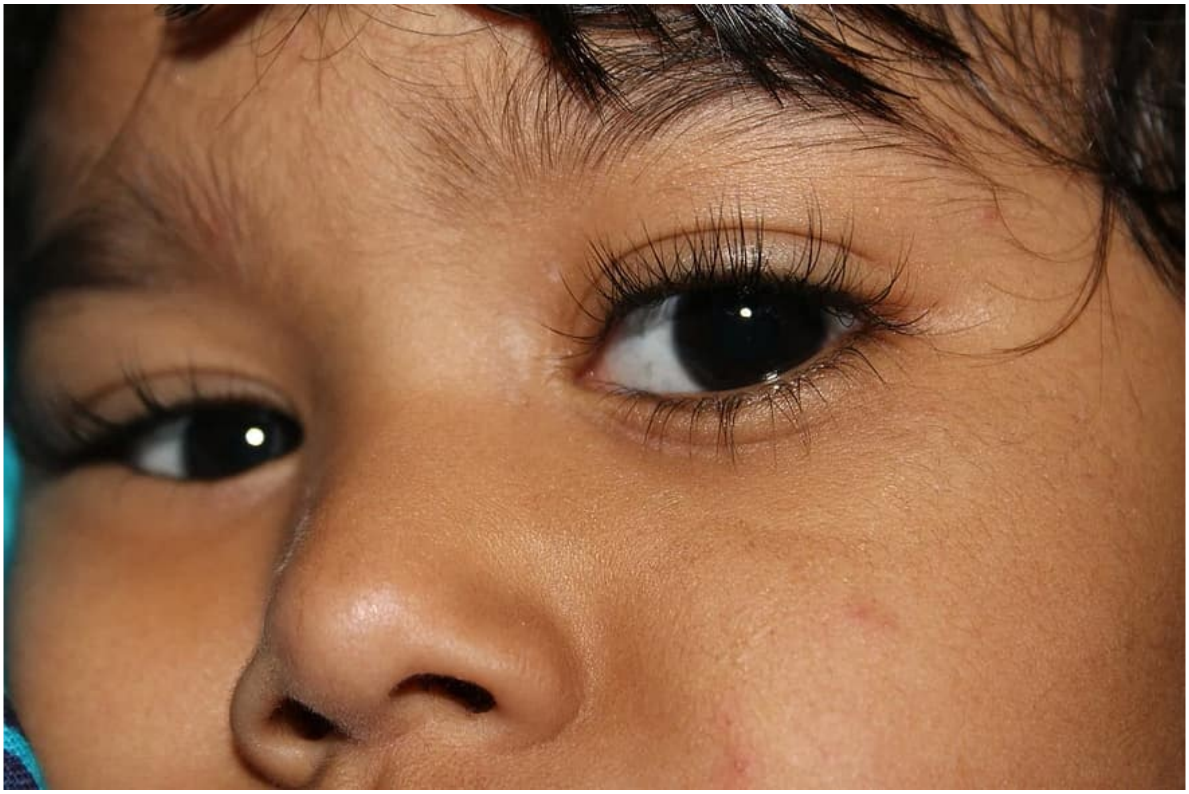

*** Continue**

◯ Yes

◯ No

Eye Research Priority Survey

Childhood-Onset Eye Disorders Research Priorities

In this section, we are asking you to indicate which research priority questions you feel are important within Childhood-Onset Eye Disorders Research. You will then be asked to rank each research question selected in order of priority.

*** Please select each research question you feel is a high priority up to a maximum of ten questions.**

- [ ] **How can the prevention, diagnosis and treatment of Cerebral Visual Impairment (CVI) in children be improved?** - *CVI is a visual impairment caused by damage to the parts of the brain that process vision. It is usually from birth but can occur later in life.*

- [ ] **How can the prevention, diagnosis and treatment of visual loss caused by prematurity be improved?** - *Prematurity (being born 3 or more weeks early) can damage an infant's vision in many different ways, often depending on how premature they are. Treatments often need to be rapid to reduce life-long visual problems.*

- [ ] **How can the early detection of visual disorders in childhood be improved?** - *Visual disorders in children need to be identified promptly so that treatments are effective (while the brain is still learning to see) and diseases affecting other parts of the body are identified early.*

- [ ] **How can the prevention, diagnosis and treatment of refractive error in children be improved?** - *Refractive error (being long-sighted, short-sighted or having astigmatism) is extremely common. It is a common cause for Amblyopia (Lazy eye). Some refractive errors (such as short-sightedness) can increase the risk of other eye conditions later in life, such as retinal detachment.*

- [ ] **How can the prevention, diagnosis and treatment of Strabismus be improved?** - *Strabismus or 'squint' (when the eyes are not aligned), is very common in children. It can be caused by other conditions and often results in Amblyopia (Lazy eye). There are many different types of strabismus and each is managed differently.*

- [ ] **How can the prevention, diagnosis and treatment of amblyopia (Lazy eye) be improved?** - *Amblyopia is a very common reason for lifelong poor vision starting in childhood. Prevention, early detection and effective treatments are key to reducing visual loss due to this common condition.*

- [ ] **How can the diagnosis and treatment of childhood cataracts be improved?** - *Cataracts in children are rare compared to adults and are often caused by genetic conditions affecting other parts of the body, besides the eyes. Surgery is much more complex and often needs to be performed in the first few weeks of life.*

- [ ] **How can the prevention, diagnosis and treatment of ocular, orbital and visual pathway tumours in children be improved?** - *Tumours (abnormal tissue lumps) in children can be caused by many things including (but not exclusively) cancers. Those affecting the eye, eye socket or visual pathway are not rare.*

- [ ] **How can the prevention, diagnosis and treatment of optic nerve disorders, including glaucoma, in children be improved?** - *Disorders of the optic nerve are not rare in children but can be more difficult to prevent, diagnose, monitor and treat, than in adults. Glaucoma is an optic nerve disorder, often related to high pressure in the eye, and one of the more common causes.*

- [ ] **How can the prevention, diagnosis and treatment of nystagmus and albinism be improved?** - *Nystagmus (uncontrolled movement of the eyes) and Albinism (a disorder of pigment production) are common causes of visual loss starting in childhood. Despite this, diagnosis can be complex and treatments are currently very limited.*

- [ ] **What improvements can be made in the assessment of visual function in children, including outcome measures for clinical studies and vision-related quality of life?** - *When treating children with visual disorders, or doing clinical studies of any type, it is important to be able to measure different aspects of vision accurately. However, this is often difficult for children.*

- [ ] **How can 'best practice' be standardised for children with rare visual disorders?** - *Many different rare diseases cause vision loss in children. Because each is individually rare, children often undergo very different patterns of care because of a lack of 'standardised' best practices.*

- [ ] **How can the diagnosis and treatment of inherited retinal disorders be improved?** - *Inherited retinal disorders are one of the most common causes of severe visual impairment in children. Diagnosis is important for good clinical care and treatments are currently lacking in most forms.*

- [ ] **How can genomic medicine be exploited to improve the prevention, diagnosis and treatment of childhood disorders of vision?** - *Genomic medicine involves testing very large amounts of DNA in individuals (and sometimes relatives), to help with preventing, diagnosing and treating diseases. It is being rapidly introduced into the standard clinical care for children with some eye diseases.*

- [ ] **How can biomarkers and bioresources be exploited to improve the prevention, diagnosis and treatment of childhood disorders of vision?** - *Biomarkers are 'biological measurements' which indicate the presence of a disease or condition. Bioresources are large collections of data from patients which may include blood sample results, heights and weights etc.*

Eye Research Priority Survey

Childhood-Onset Eye Disorders Research Priorities

In this section, we are asking you to indicate which research priority questions you feel are important within Childhood-Onset Eye Disorders Research. You will then be asked to rank each research question selected in order of priority.

*** Please could you now rank the high priority questions selected in the previous question in order of priority? The questions can be dragged and dropped or ranked with one being the highest and ten being the lowest.**

- **How can the prevention, diagnosis and treatment of Cerebral Visual Impairment (CVI) in children be improved?** - *CVI is a visual impairment caused by damage to the parts of the brain that process vision. It is usually from birth but can occur later in life.*
- **How can the prevention, diagnosis and treatment of visual loss caused by prematurity be improved?** - *Prematurity (being born 3 or more weeks early) can damage an infant's vision in many different ways, often depending on how premature they are. Treatments often need to be rapid to reduce life-long visual problems.*
- **How can the early detection of visual disorders in childhood be improved?** - *Visual disorders in children need to be identified promptly so that treatments are effective (while the brain is still learning to see) and diseases affecting other parts of the body are identified early.*
- **How can the prevention, diagnosis and treatment of amblyopia (Lazy eye) be improved?** - *Amblyopia is a very common reason for lifelong poor vision starting in childhood. Prevention, early detection and effective treatments are key to reducing visual loss due to this common condition.*
- **How can the diagnosis and treatment of childhood cataracts be improved?** - *Cataracts in children are rare compared to adults and are often caused by genetic conditions affecting other parts of the body, besides the eyes. Surgery is much more complex and often needs to be performed in the first few weeks of life.*
- **How can the prevention, diagnosis and treatment of ocular, orbital and visual pathway tumours in children be improved?** - *Tumours (abnormal tissue lumps) in children can be caused by many things including (but not exclusively) cancers. Those affecting the eye, eye socket or visual pathway are not rare.*

How can the prevention, diagnosis and treatment of optic nerve disorders, including glaucoma, in children be improved? - *Disorders of the optic nerve are not rare in children but can be more difficult to prevent, diagnose, monitor and treat, than in adults. Glaucoma is an optic nerve disorder, often related to high pressure in the eye, and one of the more common causes.*

How can the prevention, diagnosis and treatment of nystagmus and albinism be improved? - *Nystagmus (uncontrolled movement of the eyes) and Albinism (a disorder of pigment production) are common causes of visual loss starting in childhood. Despite this, diagnosis can be complex and treatments are currently very limited.*

How can the prevention, diagnosis and treatment of refractive error in children be improved? - *Refractive error (being long-sighted, short-sighted or having astigmatism) is extremely common. It is a common cause for Amblyopia (Lazy eye). Some refractive errors (such as short-sightedness) can increase the risk of other eye conditions later in life, such as retinal detachment.*

How can the prevention, diagnosis and treatment of Strabismus be improved? - *Strabismus or 'squint' (when the eyes are not aligned), is very common in children. It can be caused by other conditions and often results in Amblyopia (Lazy eye). There are many different types of strabismus and each is managed differently.*

What improvements can be made in the assessment of visual function in children, including outcome measures for clinical studies and vision-related quality of life? - *When treating children with visual disorders, or doing clinical studies of any type, it is important to be able to measure different aspects of vision accurately. However, this is often difficult for children.*

How can 'best practice' be standardised for children with rare visual disorders? - *Many different rare diseases cause vision loss in children. Because each is individually rare, children often undergo very different patterns of care because of a lack of 'standardised' best practices.*

How can the diagnosis and treatment of inherited retinal disorders be improved? - *Inherited retinal disorders are one of the most common causes of severe visual impairment in children. Diagnosis is important for good clinical care and treatments are currently lacking in most forms.*

How can genomic medicine be exploited to improve the prevention, diagnosis and treatment of childhood disorders of vision? - *Genomic medicine involves testing very large amounts of DNA in individuals (and sometimes relatives), to help with preventing, diagnosing and treating diseases. It is being rapidly introduced into the standard clinical care for children with some eye diseases.*

How can biomarkers and bioresources be exploited to improve the prevention, diagnosis and treatment of childhood disorders of vision? - *Biomarkers are 'biological measurements' which indicate the presence of a disease or condition. Bioresources are large collections of data from patients which may include blood sample results, heights and weights etc.*

Eye Research Priority Survey

Neuro-ophthalmology Research: What should the research priorities be?

An eye research priority setting exercise being undertaken through the UK Clinical Eye Research Strategy. The aim of this priority setting exercise is to update the hugely useful James Lind Alliance (JLA) Priority Setting Partnership (PSP) for Sight Loss and Vision exercise of 2013 and help guide eye research in the future.

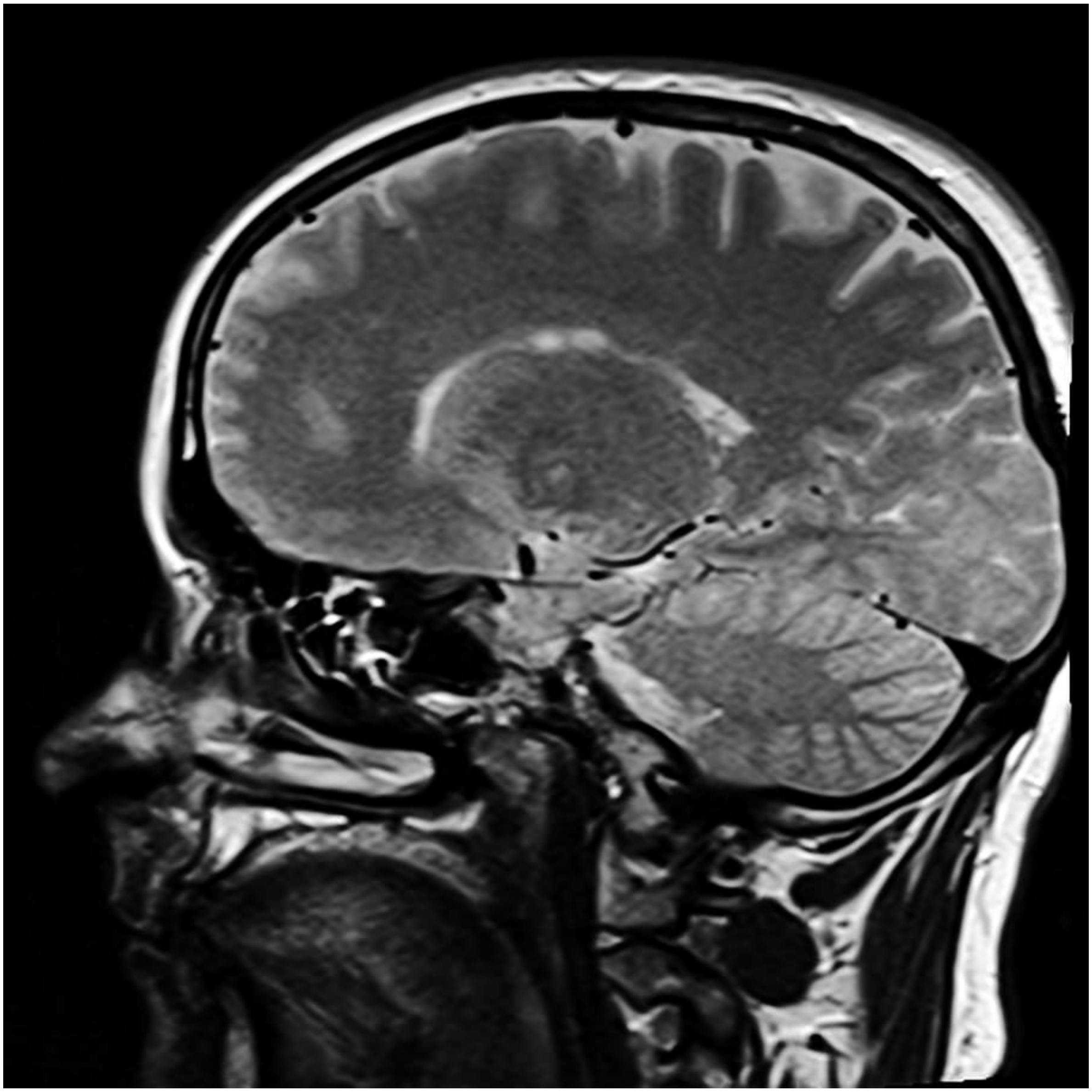

*** Continue**

- ◯ Yes
- ◯ No

Eye Research Priority Survey

Neuro-ophthalmology Research Priorities

In this section, we are asking you to indicate which research priority questions you feel are important within Neuro-ophthalmology Research. You will then be asked to rank each research question selected in order of priority.

*** Please select each research question you feel is a high priority.**

- [ ] **How can the prevention, diagnosis and treatment of hereditary optic neuropathies be improved?**- *Hereditary optic neuropathies are a group of inherited conditions which cause disease of the optic nerve which transmits visual information from the eye to the brain. They may occur in young or older people.*

- [ ] **How can the prevention, diagnosis and treatment of acquired optic neuropathies be improved?** - *Acquired optic neuropathies are a group of conditions which cause disease of the optic nerve which transmits visual information from the eye to the brain. They are not inherited and may occur in young or older people.*

- [ ] **How can the prevention, diagnosis and treatment of stroke affecting vision be improved?** - *A stroke (when blood supply to a part of the brain is interrupted) can affect many parts of the visual system and result in many forms of visual loss. It can occur in children but more typically in adults.*

- [ ] **How can the prevention, diagnosis and treatment of neurodegeneration affecting vision be improved?** - *Neurodegeneration is the progressive loss of nerve cells in the brain or nerves, for example in Alzheimer's or Parkinson's disease. It is a process of tissue damage common to many diseases affecting vision.*

- [ ] **How can the prevention, diagnosis and treatment of neuroinflammation affecting vision be improved?** - *Neuroinflammation is the damage to the brain or nerves caused by the body's own immune system. It is a process of tissue damage common to many diseases affecting vision.*

- [ ] **How can the prevention, diagnosis and treatment of intracranial tumours affecting vision be improved?** - Tumours (abnormal tissue lumps) can be caused by many things including, but not exclusively, cancers. Those occurring within the head (intracranial), commonly affect various aspects of vision

- [ ] **How can the diagnosis and treatment of traumatic brain injury (TBI) affecting vision be improved?** - *Traumatic Brain Injury (TBI) is a form of brain damage caused by a sudden injury to the head, often from trauma (such as a car accident). It is a common cause of brain injury, especially in younger adults and can affect multiple aspects of vision.*

- [ ] **How can the prevention, diagnosis and treatment of strabismus in adults be improved?** - *Strabismus or 'squint' (when the eyes are not aligned) in adults can either be present from childhood, or acquired as an adult. There are many different types of strabismus in adults and each has it's own cause, symptoms and treatment.*

- [ ] **How can biomarkers and bio-resources be exploited to improve the prevention, diagnosis, monitoring and treatment of adult neuro-ophthalmic disorders?** - *Biomarkers are 'biological measurements' which indicate the presence of a disease or condition. Bio-resources are large collections of data from patients which may include blood sample results, heights and weights etc.*

Eye Research Priority Survey

Neuro-ophthalmology Research Priorities

In this section, we are asking you to indicate which research priority questions you feel are important within Neuro-ophthalmology Research. You will then be asked to rank each research question selected in order of priority.

*** Please could you now rank the high priority questions selected in the previous question in order of priority? The questions can be dragged and dropped or ranked with one being the highest and nine being the lowest.**

- **How can the prevention, diagnosis and treatment of hereditary optic neuropathies be improved?**- *Hereditary optic neuropathies are a group of inherited conditions which cause disease of the optic nerve which transmits visual information from the eye to the brain. They may occur in young or older people.*
- **How can the prevention, diagnosis and treatment of acquired optic neuropathies be improved?** - *Acquired optic neuropathies are a group of conditions which cause disease of the optic nerve which transmits visual information from the eye to the brain. They are not inherited and may occur in young or older people.*
- **How can the prevention, diagnosis and treatment of stroke affecting vision be improved?** - *A stroke (when blood supply to a part of the brain is interrupted) can affect many parts of the visual system and result in many forms of visual loss. It can occur in children but more typically in adults.*
- **How can the prevention, diagnosis and treatment of neurodegeneration affecting vision be improved?** - *Neurodegeneration is the progressive loss of nerve cells in the brain or nerves, for example in Alzheimer's or Parkinson's disease. It is a process of tissue damage common to many diseases affecting vision.*
- **How can the prevention, diagnosis and treatment of neuroinflammation affecting vision be improved?** -*Neuroinflammation is the damage to the brain or nerves caused by the body's own immune system. It is a process of tissue damage common to many diseases affecting vision.*
- **How can the prevention, diagnosis and treatment of intracranial tumours affecting vision be improved?** - Tumours (abnormal tissue lumps) can be caused by many things including, but not exclusively, cancers. Those occurring within the head (intracranial), commonly affect various aspects of vision
- **How can the diagnosis and treatment of traumatic brain injury (TBI) affecting vision be improved?** - *Traumatic Brain Injury (TBI) is a form of brain damage caused by a sudden injury to the head, often from trauma (such as a car accident). It is a common cause of brain injury, especially in younger adults and can affect multiple aspects of vision.*
- **How can the prevention, diagnosis and treatment of strabismus in adults be improved?** - *Strabismus or 'squint' (when the eyes are not aligned) in adults can either be present from childhood, or acquired as an adult. There are many different types of strabismus in adults and each has it's own cause, symptoms and treatment.*
- **How can biomarkers and bio-resources be exploited to improve the prevention, diagnosis, monitoring and treatment of adult neuro-ophthalmic disorders?** - *Biomarkers are 'biological measurements' which indicate the presence of a disease or condition. Bio-resources are large collections of data from patients which may include blood sample results, heights and weights etc.*

Eye Research Priority Survey

Uveitis Research: What should the research priorities be?

An eye research priority setting exercise being undertaken through the UK Clinical Eye Research Strategy. The aim of this priority setting exercise is to update the hugely useful James Lind Alliance (JLA) Priority Setting Partnership (PSP) for Sight Loss and Vision exercise of 2013 and help guide eye research in the future.

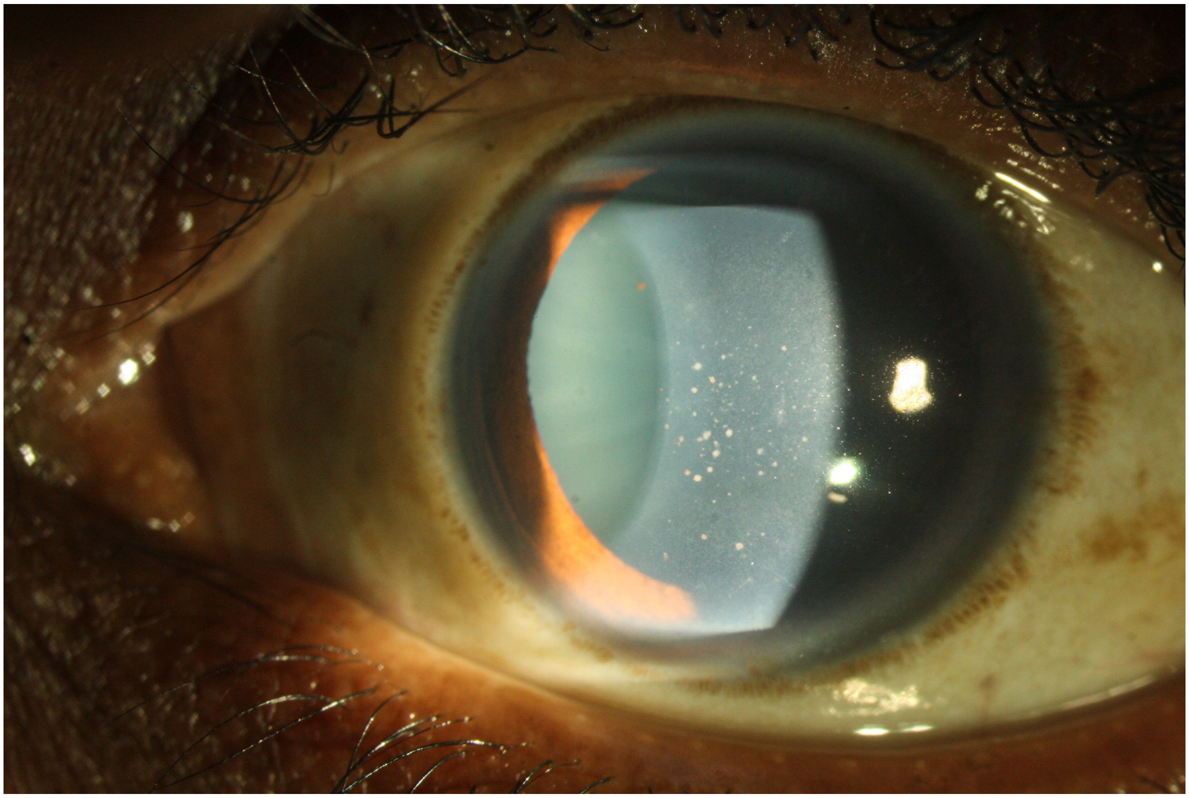

*** Continue**

◯ Yes

◯ No

Eye Research Priority Survey

Uveitis Research Priorities

In this section, we are asking you to indicate which research priority questions you feel are important within Uveitis Research. You will then be asked to rank each research question selected in order of priority.

* **Please select each research question you feel is a high priority.**

- [ ] What are the most effective treatments for ocular and orbital inflammatory diseases?
- [ ] Which licensed treatments for systemic inflammatory diseases (but not for uveitis) are effective in inflammatory eye disease?
- [ ] What is the cause and most effective medical management for Thyroid Eye Disease?
- [ ] What causes uveitis or scleritis in isolated ocular disease and in systemic disease with associated disease?
- [ ] What causes relapse in ocular inflammatory disease and how long should we treat patients?
- [ ] What are the most effective biomarkers (imaging / non-imaging) to predict relapse or monitor for disease progression in ocular or orbital inflammatory disease?
- [ ] What are the best ways to personalise treatment in uveitis and scleritis?
- [ ] What are the most effective scoring systems and clinical outcome measures (imaging / non-imaging) of disease and treatment response in ocular or orbital inflammatory disease?
- [ ] How can we improve ways to diagnose infectious uveitis?

Eye Research Priority Survey

Uveitis Research Priorities

In this section, we are asking you to indicate which research priority questions you feel are important within Uveitis Research. You will then be asked to rank each research question selected in order of priority.

*** Please could you now rank the high priority questions selected in the previous question in order of priority? The questions can be dragged and dropped or ranked with one being the highest and nine being the lowest.**

- What are the most effective treatments for ocular and orbital inflammatory diseases?
- Which licensed treatments for systemic inflammatory diseases (but not for uveitis) are effective in inflammatory eye disease?
- What is the cause and most effective medical management for Thyroid Eye Disease?
- What causes uveitis or scleritis in isolated ocular disease and in systemic disease with associated disease?
- What causes relapse in ocular inflammatory disease and how long should we treat patients?
- What are the most effective biomarkers (imaging / non-imaging) to predict relapse or monitor for disease progression in ocular or orbital inflammatory disease?
- What are the best ways to personalise treatment in uveitis and scleritis?
- What are the most effective scoring systems and clinical outcome measures (imaging / non-imaging) of disease and treatment response in ocular or orbital inflammatory disease?
- How can we improve ways to diagnose infectious uveitis?

Eye Research Priority Survey

Final Comments

You can type into the comment box any issues that you think need highlighting. Please state which research priority questionnaire you are referring to.

You may also leave your contact details if you wish to take part in the next step of the process (framing research priorities into research questions leading to appropriate study designs that initiate new research projects).

We would be grateful if you could inform us where you have heard about this survey

Eye Research Priority Survey

Thank you for your participation

*** Would you like to complete another survey that covers a different topic of eye research?**

◯ Yes

◯ No
