# Supplementary material for: The UK clinical eye research strategy: refreshing research priorities for clinical eye research in the UK
Source: Eye (Lond). 2024 May 29;38(10):1947–57. doi: 10.1038/s41433-024-03049-6 (PMC11226710; doi:10.1038/s41433-024-03049-6)
Supplement: Supplementary file 2 — Appendix 2- Dissemination Strategy [file 41433_2024_3049_MOESM2_ESM.docx]

**UK Clinical Eye Research Strategy**

**Dissemination Distribution List**

| **Stakeholder Group** |
| --- |
| UK Clinical Eye Research Strategy  <https://ukeyeresearchstrategy.ac.uk> |
| NIHR  <https://www.nihr.ac.uk/explore-nihr/specialties/ophthalmology.htm> |
| NIHR Ophthalmology Specialty Group  <https://www.nihr.ac.uk/explore-nihr/specialties/ophthalmology.htm> |
| James Lind Alliance  <https://www.jla.nihr.ac.uk> |
| Macular Society  <https://www.macularsociety.org> |
| Fight for Sight  <https://www.fightforsight.org.uk> |
| Moorfields Eye Charity  <https://moorfieldseyecharity.org.uk> |

| Retina UK  <https://retinauk.org.uk> |
| --- |
| RNIB  <https://www.rnib.org.uk> |
| The Royal College of Ophthalmologists  <https://www.rcophth.ac.uk> |
| The College of Optometrists  <https://www.college-optometrists.org> |
| College of Optometrists  <https://lookafteryoureyes.org> |
| NETSCC [NIHR Evaluation, Trials and Studies Coordinating Centre](https://www.southampton.ac.uk/netscc/index.page?) – all the main NIHR funding bodies |
| Acuity Foundation Ireland |
| Association of Blind Asians  [info@abaleeds.org.uk](mailto:info@abaleeds.org.uk)[https://abaleeds.org.uk/](https://www.google.com/url?q=https://abaleeds.org.uk/&source=gmail-imap&ust=1649321062000000&usg=AOvVaw2d5pdC-4kvyLicLAec5NGP) |
| Action for Blind People |
| Age UK |
| Association of British Dispensing Opticians |
| Behcet’s Syndrome Society |
| Blind Veterans UK |
| British and Irish Orthoptic Society |
| British Thyroid Foundation |
| Contact a Family |
| Eyecare Trust |
| Guide Dogs |
| Glaucoma UK |
| Juvenile Diabetes Research Foundation |
| Keratoconus Group |
| Kingston Association for the Blind |
| Micro and Anophthalmic Children’s Society |
| National Blind Children’s Society |
| National Federation of the Blind of the UK |
| NRS Ophthalmology Scotland |
| Ocumel |
| One Clear Vision |
| Organisation of Blind African Caribbeans |
| Polymyalgia Rheumatica and Giant Cell Arteritis UK (PMRGCAUKK) |
| RP Fighting Blindness |
| Royal College of Nursing |
| Thomas Pocklington Trust |
| Thyroid Eye Disease Charitable Trust |
| UK & Eire Glaucoma Society |
| Visibility |
| Vision Care for Homeless People  [info@vchp.org.uk](mailto:info@vchp.org.uk)[https://www.visioncarecharity.org/](https://www.google.com/url?q=https://www.visioncarecharity.org/&source=gmail-imap&ust=1649321062000000&usg=AOvVaw1HmIrMqo_iU1de6_qg0fWT) |
| Wales Vision Strategy Group |
| Waltham Forest Low Vision Forum |
| West of England School and College |
| UKNOS - [https://uknos.com/](https://www.google.com/url?q=https://uknos.com/&source=gmail-imap&ust=1649260161000000&usg=AOvVaw0wG6sQDBNHG5neEHNLk0oG)  Online information resources such as: [https://www.sightadvicefaq.org.uk/newly-diagnosed-registration/newly-diagnosed/eclo](https://www.google.com/url?q=https://www.sightadvicefaq.org.uk/newly-diagnosed-registration/newly-diagnosed/eclo&source=gmail-imap&ust=1649260161000000&usg=AOvVaw2kx4pkU_Tm4uPBGf2DJaAu)  BIOS - [https://www.orthoptics.org.uk/](https://www.google.com/url?q=https://www.orthoptics.org.uk/&source=gmail-imap&ust=1649260161000000&usg=AOvVaw2oFdKA4Wb09vvoBoE0y9cm)  Seeability - [https://www.seeability.org/](https://www.google.com/url?q=https://www.seeability.org/&source=gmail-imap&ust=1649260161000000&usg=AOvVaw3H0xga6GsmQgyYZ6Rrfnif)  CVI Scotland and CVI society - [https://cvisociety.org.uk/](https://www.google.com/url?q=https://cvisociety.org.uk/&source=gmail-imap&ust=1649260161000000&usg=AOvVaw2l4tXb9XliGuOfT-OyJllT)  Angel Eyes (Northern Ireland) - [https://www.angeleyesni.org/](https://www.google.com/url?q=https://www.angeleyesni.org/&source=gmail-imap&ust=1649260161000000&usg=AOvVaw2E7J-nJFzNLlxF7ZKwa9_e)  Guide dogs - [https://www.guidedogs.org.uk](https://www.google.com/url?q=https://www.guidedogs.org.uk/how-you-can-help/donating/sponsor-a-puppy/?gclid%3DCj0KCQjw_4-SBhCgARIsAAlegrUCcfxKonrVkfixccbcDlpVfBHFHTUxts0BG8MPrESmxC5WEpSsBcIaApnrEALw_wcB%26gclsrc%3Daw.ds&source=gmail-imap&ust=1649260161000000&usg=AOvVaw1H4SZWrOWj8YR7-5Ut4IVb)  Stroke association - [https://www.stroke.org.uk/](https://www.google.com/url?q=https://www.stroke.org.uk/&source=gmail-imap&ust=1649260161000000&usg=AOvVaw2aaFCdRDeB6RlsgBrxkdys)  Headway – [https://www.headway.org.uk/](https://www.google.com/url?q=https://www.headway.org.uk/&source=gmail-imap&ust=1649260161000000&usg=AOvVaw0cWTlYMwTpm8Cgeizw8WNC)  The Brain charity - [https://www.thebraincharity.org.uk/](https://www.google.com/url?q=https://www.thebraincharity.org.uk/&source=gmail-imap&ust=1649260161000000&usg=AOvVaw0hekkXuW8z5kEgk-AYnJ_p)  UK support group for Stargardt’s and juvenile central vision loss [https://www.facebook.com/groups/198176107521426/?ref=share](https://www.google.com/url?q=https://www.facebook.com/groups/198176107521426/?ref%3Dshare&source=gmail-imap&ust=1649260161000000&usg=AOvVaw3o37Vdz-iWaIjaketXFYrR)  New UK charity for Stargardt’s [https://stargardtsconnected.org.uk/](https://www.google.com/url?q=https://stargardtsconnected.org.uk/&source=gmail-imap&ust=1649260161000000&usg=AOvVaw1rd7A_CY18C2CPF0zaZi1q)  Glaucomarize, [http://www.glaucomarize.org/](https://www.google.com/url?q=http://www.glaucomarize.org/&source=gmail-imap&ust=1649260161000000&usg=AOvVaw22TvvcK2m3VbRmFlKygzj-)  Glaucoma Association Support Groups [https://www.glaucoma-association.com/about-the-iga/what-we-do/groups-in-your-area](https://www.google.com/url?q=https://www.glaucoma-association.com/about-the-iga/what-we-do/groups-in-your-area&source=gmail-imap&ust=1649260161000000&usg=AOvVaw0F9twszTMmOEKYwpvoo6Dl)  Birdshot Uveitis UK based and international <https://www.facebook.com/birdshotuveitis/> and [https://www.facebook.com/groups/Birdshot/](https://www.google.com/url?q=https://www.facebook.com/groups/Birdshot/&source=gmail-imap&ust=1649260161000000&usg=AOvVaw1v0DOWdOd1dqRziGCHyMgQ)  The Nystagmus Family Foundation <https://www.facebook.com/groups/924377374279223/> and Nystagmus Network  [https://www.facebook.com/NystagmusNetwork/?__tn__=%2Cd%2CP-R&eid=ARDN9D0tuCPt7NECC1b3O6Webr3p5M6UyQCPGGmP7lsgJaI6IFc2hhiQ4pj2M9pJag9sJim6CFcbqr9s](https://www.google.com/url?q=https://www.facebook.com/NystagmusNetwork/?__tn__%3D%252Cd%252CP-R%26eid%3DARDN9D0tuCPt7NECC1b3O6Webr3p5M6UyQCPGGmP7lsgJaI6IFc2hhiQ4pj2M9pJag9sJim6CFcbqr9s&source=gmail-imap&ust=1649260161000000&usg=AOvVaw3IfRPbMIyooXxz_GJIqiUx)  UK Macular Degeneration Friendship Group [https://www.facebook.com/groups/589067591220411/](https://www.google.com/url?q=https://www.facebook.com/groups/589067591220411/&source=gmail-imap&ust=1649260161000000&usg=AOvVaw3m0qb1izzRO-2uw5gMzkO2)  Macular Degeneration Community [https://www.facebook.com/groups/1674702946079370/?ref=br_rs](https://www.google.com/url?q=https://www.facebook.com/groups/1674702946079370/?ref%3Dbr_rs&source=gmail-imap&ust=1649260161000000&usg=AOvVaw1_eiO94QXCXgA04Yzr0MGn)  Leber Congenital Amaurosis – LCA [https://www.facebook.com/Leber-Congenital-Amaurosis-LCA-240567533163063/](https://www.google.com/url?q=https://www.facebook.com/Leber-Congenital-Amaurosis-LCA-240567533163063/&source=gmail-imap&ust=1649260161000000&usg=AOvVaw25hU20UoQ4gus0WG1wWm4I)  [https://www.facebook.com/pg/LebersCongenitalAmaurosis/about/?ref=page_internal](https://www.google.com/url?q=https://www.facebook.com/pg/LebersCongenitalAmaurosis/about/?ref%3Dpage_internal&source=gmail-imap&ust=1649260161000000&usg=AOvVaw2PBQGbNxXrg8KR5ZP37CTU)  Childhood Eye Cancer Trust [https://chect.org.uk/](https://www.google.com/url?q=https://chect.org.uk/&source=gmail-imap&ust=1649260161000000&usg=AOvVaw0lPqHXkhgECU2cuON2bNAq)  Young Epilepsy [https://www.youngepilepsy.org.uk/](https://www.google.com/url?q=https://www.youngepilepsy.org.uk/&source=gmail-imap&ust=1649260161000000&usg=AOvVaw1SxZxTX_YpSrAg4K1Unosx)  The Pocklington Trust - [https://www.pocklington-trust.org.uk/](https://www.google.com/url?q=https://www.pocklington-trust.org.uk/&source=gmail-imap&ust=1649260161000000&usg=AOvVaw3tNiiNiL3O8lhUiBYcNUam)  Generation R Eye-YPAG (Young Patients Advisory Group) [https://generationr.org.uk/eyeypag-evaluation](https://www.google.com/url?q=https://generationr.org.uk/eyeypag-evaluation&source=gmail-imap&ust=1649260161000000&usg=AOvVaw1hBu7sauj3YTH9u8kETztV)  BCCIG and BCVIS study group members – |
| Bird Shot Charity |
| BIPOSA |
| BMJ Ophthalmology |
| Bradford Ophthalmology Research Network (BORN) |
| Cochrane Eyes and Vision Group |
| Cure CHM |
| Eye Journal |
| Eye News |
| Gift of Sight |
| Industry Vision Group (IVG)  The Industry Vision Group (IVG) is a cross-industry alliance of five companies, AbbVie Ltd, Bayer, Novartis, Roche and Santen. |
| Insight Eye hub |
| Leeds Optical committee |
| Macular Degeneration |
| National Eye Health Week (19-25 Sept 2022) |
| NICE |
| NIHR Moorfields BRC and their PPI leads |
|  |
| Nystagmus Network |
| Optometry Today |
| Sight Research UK |
| The AOP  [Advanced Ophthalmologic Practice](https://www.aopcongress.com/en) |
| The General Optical Council |
| The Ophthalmologist |
| UCL Institute of Ophthalmology |
| Uveitis CSG  https://www.uveitisstudygroup.org |
| VERI |
| Visionary UK |
| Wales Council Blind |
| Wellcome Trust |
| Women in Vison UK |
| Devolved Nations societies/Prof groups |
