# Supplementary material for: The UK clinical eye research strategy: refreshing research priorities for clinical eye research in the UK
Source: Eye (Lond). 2024 May 29;38(10):1947–57. doi: 10.1038/s41433-024-03049-6 (PMC11226710; doi:10.1038/s41433-024-03049-6)
Supplement: Supplementary file 3 — Appendix 3 - Contributions by Authors [file 41433_2024_3049_MOESM3_ESM.docx]

**Appendix 3: Contributions by Authors**

## NIHR Ophthalmology Specialty Group:

## Providing data or critical feedback on data sources

Nick AV Beare, Rupert RA Bourne, Christiana Dinah, Manjo Doug, Richard P Gale, Louise Gow, Chris Hammond, Padraig J Mulholland, Praveen J Patel, George M Saleh, Peter Scanlon, Brinda Shah

## Developing methods or computational machinery

Rupert RA Bourne, Manjo Doug, Geraldine V Hoad, Praveen J Patel, George M Saleh

## Providing critical feedback on methods or results

Ejaz Ansari, Rupert RA Bourne, , Francesca Cordeiro, Christiana Dinah, Manjo Doug, Richard P Gale, Faruque Ganchi, Louise Gow, Geraldine V Hoad, Anthony King, Andrew J Lotery, Padraig J Mulholland, Praveen J Patel, Fiona J Rowe, George M Saleh, Julie Silvestri, Velota Sung, Andrew J Tatham, Marcela Vortruba

## Drafting the work or revising it critically for important intellectual content

Ejaz Ansari, Nick AV Beare, Rupert RA Bourne, Manjo Doug, Susan Downes, Richard P Gale, Faruque Ganchi, Chris Hammond, Geraldine V Hoad, Anthony King, Andrew J Lotery, Padraig J Mulholland, Fiona J Rowe, George M Saleh, Julie Silvestri, Andrew J Tatham, Marta M Urgate, Deepali Varma, Marcela Vortruba

## Managing the estimation or publications process

## Rupert RA Bourne, Manjo Doug

Executive Group of UK Clinical Eye Research Strategy:

## Providing data or critical feedback on data sources

## Rupert RA Bourne, , Michael Bowen, Manjo Doug, , Richard P Gale, Louise Gow, Praveen J Patel, George M Saleh, James E Self, Srilakshmi M Sharma, Sobha Sivaprasad

1. Developing methods or computational machinery

Rupert RA Bourne, Catey Bunce, Roxanne Crosby, Manjo Doug, Geraldine V Hoad, Ailish Murray, Praveen J Patel, George M Saleh, James E Self, Sobha Sivaprasad

## Providing critical feedback on methods or results

Augusto Azuara Blanco, Rupert RA Bourne, Catey Bunce, Michael Bowen, Manjo Doug, Faruque Ganchi, Richard P Gale, Louise Gow, Geraldine V Hoad, Praveen J Patel, George M Saleh, James E Self, Srilakshmi M Sharma, Sobha Sivaprasad

## Drafting the work or revising it critically for important intellectual content

Augusto Azuara Blanco, Rupert RA Bourne, Michael Bowen, Catey Bunce, Manjo Doug, Faruque Ganchi, Richard P Gale, Geraldine V Hoad, George M Saleh, James E Self, Srilakshmi M Sharma, Sobha Sivaprasad, Deepali Varma

## Managing the estimation or publications process

## Augusto Azuara Blanco, Rupert RA Bourne, Manjo Doug, Sobha Sivaprasad
